# Neuropixels Opto: combining high-resolution electrophysiology and optogenetics

**DOI:** 10.1038/s41592-026-03076-z

**Published:** 2026-06-01

**Authors:** Anna A. Lakunina, Karolina Z. Socha, Alexander E. Ladd, Anna J. Bowen, Susu Chen, Jennifer Colonell, Anjal Doshi, Bill Karsh, Michael Krumin, Pavel Kulik, Anna J. Li, Pieter Neutens, John O’Callaghan, Meghan Olsen, Jan Putzeys, Charu Bai Reddy, Harrie A. C. Tilmans, Sara Vargas, Marleen Welkenhuysen, Zhiwen Ye, Michael Häusser, Christof Koch, Jonathan T. Ting, Barundeb Dutta, Timothy D. Harris, Nicholas A. Steinmetz, Karel Svoboda, Joshua H. Siegle, Matteo Carandini

**Affiliations:** 1 Allen Institute for Neural Dynamics, Seattle, WA USA; 2 UCL Institute of Ophthalmology, University College London, London, UK; 3 Department of Neurobiology and Biophysics, University of Washington, Seattle, WA USA; 4 Janelia Research Campus, Howard Hughes Medical Institute, Ashburn, VA USA; 5 Department of Biomedical Engineering, Johns Hopkins University, Baltimore, MD USA; 6 IMEC, Leuven, Belgium; 7 Allen Institute for Brain Science, Seattle, WA USA; 8 Wolfson Institute for Biomedical Research, University College London, London, UK; 9 Allen Institute MindScope Program, Seattle, WA USA

**Keywords:** Extracellular recording, Neurophysiology, Optogenetics

## Abstract

High-resolution extracellular electrophysiology is the gold standard for recording spikes from distributed neural populations and is especially powerful when combined with optogenetics for manipulation of specific cell types with high temporal resolution. We integrated these approaches into prototype Neuropixels Opto probes, which combine electronic and photonic circuits. These devices pack 960 electrical recording sites and two sets of 14 light emitters onto a 70-μm-wide, 1-cm-long shank, allowing spatially addressable optogenetic stimulation with blue and red light. In mouse cortex, Neuropixels Opto probes delivered high-quality recordings together with spatially addressable optogenetics, differentially activating or silencing neurons at distinct cortical depths. In the mouse striatum and other deep structures, Neuropixels Opto probes delivered efficient optotagging, facilitating the identification of two cell types in parallel. Neuropixels Opto probes represent a promising tool for recording, identifying and manipulating neuronal populations.

## Abstract (web summary)

Neuropixels Opto probes combine high-density recording sites with optical waveguide-based light emission sites for simultaneous recording of neuronal activity and optogenetic stimulation with blue and/or red light. As demonstrated in the mouse brain, these probes allow the activation or silencing of defined groups of neurons while acquiring high-quality recordings.

## Main

Understanding brain function requires recording from myriad neurons, identifying them and manipulating their activity. For large-scale recordings, an ideal method is extracellular electrophysiology using high-density electrodes such as Neuropixels probes^1,2^. For neuron identification^3–5^ and manipulation^6–9^, in turn, a leading method is optogenetics.

Electrophysiology and optogenetics are particularly powerful when paired with each other^10–12^. By combining them, one can test the causal role of specific neural populations by activating or inactivating those populations while recording the effects on neural activity^7,13–16^. One can also identify whether the recorded neurons belong to a genetic class of interest by ‘optotagging’ (refs. ^3–5^), that is, inducing this class to express an opsin and stimulating it with light. Optotagging is critical for connecting the wealth of knowledge about the gene expression, morphology and connectivity of different cell classes to their function.

Optogenetics, however, depends critically on delivering light with sufficient intensity and spatial resolution, potentially deep in the brain. This is difficult in brain tissue, which scatters and absorbs light. It commonly requires inserting additional devices for light delivery, such as optical fibers, waveguides or microLED arrays. Existing approaches, however, have limited spatial resolution or light intensity, are invasive and require a separate device for recording.

Thus, there is great interest in combining recording and light emission into a single ‘optrode’; however, existing solutions have few recording sites or limited light intensity. Early optrodes integrated electrodes with optical fibers^17–21^, yielding few emitters. More emitters were enabled by microLEDs^22–27^. However, miniaturized microLEDs have low efficiency (1–3%); hence, even at moderate light intensities, they increase brain temperature^28,29^ by 0.5–1.5 °C (refs. ^25,30^). Thus, they deliver only low light intensities or duty ratios^31^.

To resolve these limitations, we combined Neuropixels recording technology with on-chip photonic waveguides that route high-intensity light down the shank of the probe. Light is generated outside the brain and routed by on-chip photonic waveguides^32–36^. This design enables dual-color illumination across a 1.4-mm span in parallel to voltage readout from close to 1,000 selectable recording sites per shank with on-board amplification and digitization^1,2^. The resulting prototype device, called Neuropixels Opto, integrates high-resolution electrophysiology and optogenetics.

## Results

Neuropixels Opto integrates electronics and photonics to simultaneously record signals from 384 of 960 recording sites and emit light from two sets of 14 emitters. The two sets of light emitters allow dual-color optogenetics with blue and red light, making it possible to address two genetically defined neural populations in parallel. For the red light, we chose a wavelength of 638 nm to excite highly effective red-sensitive opsins such as Chrimson^37^ and ChRmine^38^. This wavelength avoids the peaks of absorption by oxygenated blood^39^ at 420 nm and 540–580 nm, thus enhancing the penetration of light into tissue. For the blue light, we set the wavelength to 450 nm (rather than the more common 473 nm) to efficiently activate Channelrhodopsin-2 (ChR2)^40^ and its variants while reducing the activation of the red-sensitive opsins.

### Probe design

**Fig. 1:**
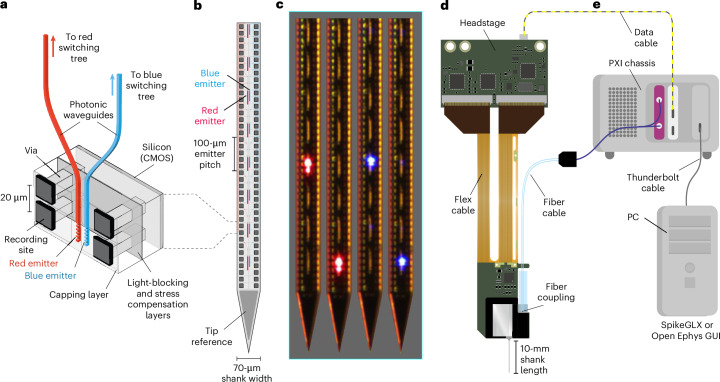
Design of the prototype Neuropixels Opto probe. **a**, Cross-section of the Neuropixels Opto probe shank, showing the TiN recording sites (connected with a ‘via’ to the silicon CMOS layer) and the SiN photonic waveguides ending in the emitters (grating couplers). **b**, Layout of recording sites and dual-color emitters. **c**, Photos of a probe shank with two red and two blue emitters delivering light in succession. **d**, Device package. **e**, Neuropixels Opto system architecture, with PXI modules for data acquisition (white) and light delivery (purple).

**Extended Data Fig. 1:**
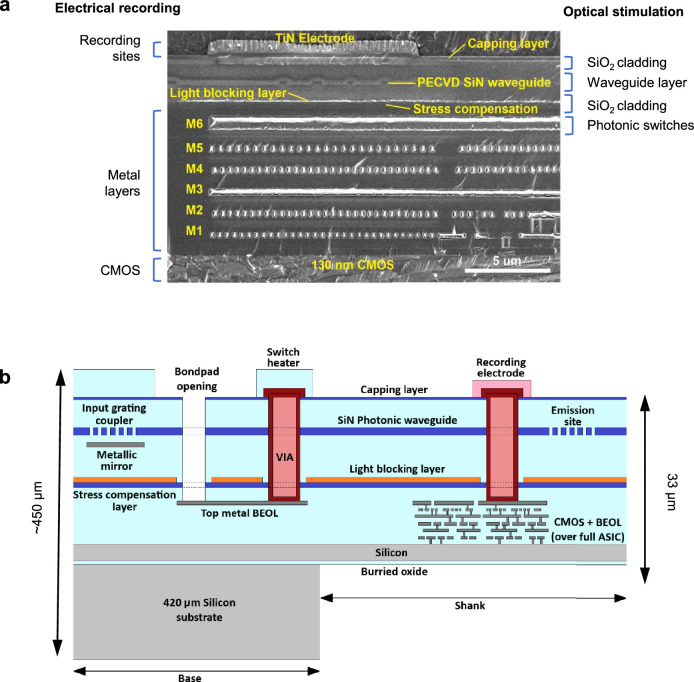
The integrated CMOS/Photonics platform. **a**. Cross-section of the probe shank obtained with a scanning electron microscope (SEM) showing the monolithically integrated platform combining the CMOS platform (for electrical recording) and Si_x_N_y_-based photonics (for optical stimulation). *Left labels*: layers used for the recording sites and readout IC, based on the 130-nm SOI CMOS Aluminum back-end-of-line process (Al BEOL) that was developed for the Neuropixels 1.0 probe. They point to the 130-nm CMOS layer (*bottom*), the 6 Aluminum metal layers (*middle*) and the Titanium nitride electrode (*top*). *Right labels*: the photonic modules that were added between the M6 metal layer of the CMOS BEOL and the recording electrode to enable optical stimulation. The photonics also use the M6 metal layer for controlling the photonic switches that are used to select the optical emitter. **b**. Schematic of the probe build-up, illustrating the photonic modules that were added to the CMOS platform, which include: grating couplers handling the light coupling from a fiber to the chip; an underlying reflector layer (metallic mirror); a Si_x_N_y_ photonic waveguide module; a full TiN/Al/TiN light blocking layer that is only interrupted for the VIA and bond pads, to protect the CMOS circuits from laser light scattered by the photonic waveguides; a heater module to make thermo-optical switches; a stress compensation layer to cancel the stress induced by the extra photonics layers, keeping the shank tip deflection within the spec of ±200 μm; and a deeper VIA etch to contact the heaters and electrodes.

Neuropixels Opto probes monolithically integrate complementary metal–oxide–semiconductor (CMOS) circuits for electrical recording with photonics for optical stimulation (Fig. 1a and Extended Data Fig. 1). An integrated silicon nitride (SiN) photonics layer provides photonic waveguides that route red and blue light to programmable emitters. The waveguides are fabricated in 150-nm-thick SiN and are routed to the distal end of the shank. To couple out the light from the waveguides to the emitters and distribute it perpendicular to the probe, we used higher-order, apodized Bragg grating couplers^41–44^ designed to spread the light over multiple diffraction peaks. The photonics layer lies above the CMOS platform designed for Neuropixels 1.0 probes^1^ (a 130-nm silicon-on-insulator CMOS Al process with six metal layers and titanium nitride (TiN) recording sites).

This design posed two challenges. First, the addition of photonics can cause the probe shank to bend. We addressed this challenge by depositing a SiN compensation layer and a SiN capping layer (Fig. 1a and Extended Data Fig. 1), reducing tip deflection to <200 μm. Second, scattered light from the photonic waveguides can interact with the CMOS circuitry, which is sensitive to light, increasing noise levels or introducing recording artifacts. To prevent light from reaching the CMOS circuits, we added a TiN/Al-based light-blocking layer (Fig. 1a and Extended Data Fig. 1), keeping it as thin as possible to minimize shank bending and thickness.

The 14 × 2 emitters (16–25 μm^2^) are arranged on the center axis of the shank and are spaced 100 μm apart, covering 1.5 mm from the shank’s tip (Fig. 1b,c). The recording site array has a similar density to Neuropixels 1.0 (ref. ^1^), with 960 TiN recording sites (12 × 12 μm^2^) separated vertically by 20 μm. The sites are arrayed in two vertical columns as in Neuropixels 2.0 (ref. ^2^), spaced 48 μm apart. The shank is 10 mm long, 70 μm wide and 33 μm thick (9 μm thicker than Neuropixels 1.0 probes). As in Neuropixels 1.0 probes, signals from each recording site are split into bands for action potentials (APs; 0.3–10 kHz, digitized at 30 kHz) and local field potentials (LFPs; <1 kHz, digitized at 2.5 kHz).

**Extended Data Fig. 2:**
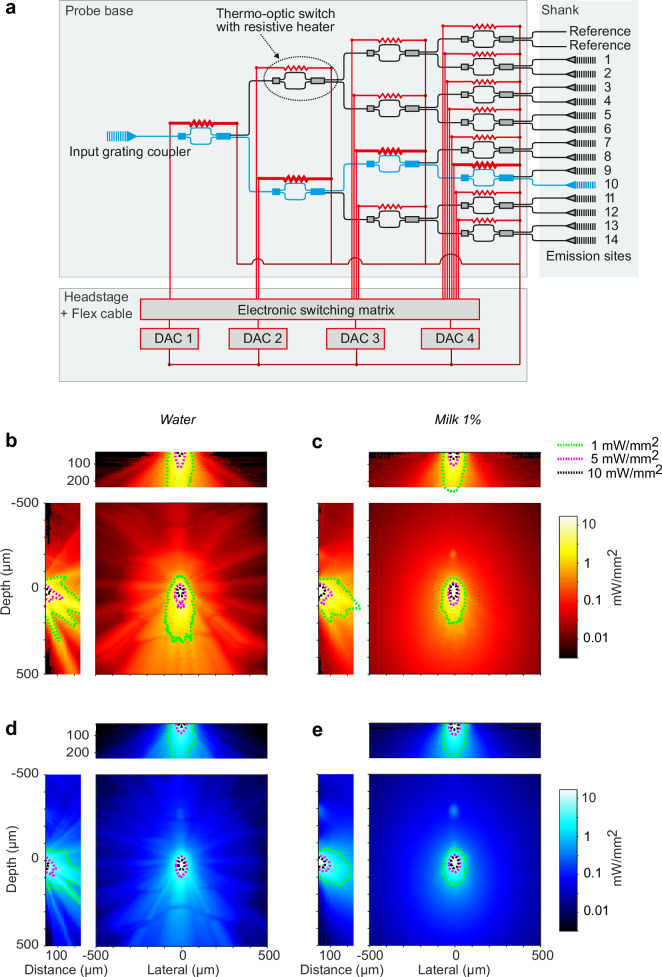
Light switching tree and optical characterization. **a**. Schematic of switching tree architecture, which distributes light from a single input grating coupler to one of fourteen waveguides on the shank. A network of 15 thermo-optic switches is controlled by four digital-to-analog converters (DACs), allowing light to be routed to each emitter, or to reference sites used for calibration. Each probe contains a separate switching tree for blue and red light. **b**. Maximal intensity projections of the 3D distribution of light emitted by a red emitter, when the probe was immersed in water. Contours show intensity levels at 1, 5, and 10 mW/mm^2^. **c**. Same, when the probe was immersed in an optical phantom (1% milk) whose estimated reduced scattering coefficient ${\mu }_{s}^{{\prime} }$ (23 cm^−1^) is in the range measured in rodent gray matter (20–30 cm^−1^, see Methods). **d, e**. Same as **b, c**, for a blue emitter.

The light is generated by two fiber-coupled lasers at 450 and 638 nm, connected to the probe by grating couplers and routed to the emitters by two photonic switching trees. There are eight grating couplers: two to couple the light from the two fibers and the others for active alignment of the fiber block and measurement of coupling losses. The light of each color is routed to the desired emitters by a programmable photonic binary switching tree (Extended Data Fig. 2a). The switches are Mach–Zehnder interferometers with thermal phase shifters based on the thermo-optic effect. After calibration, they are the optical equivalent of a toggle switch. With four levels, the tree can specify 2^4^ = 16 outputs and, thus, address the 14 emitters independently. The current version of the probe allows one emitter per color to be on at a time but any combination is possible in principle.

This switching tree was calibrated once, after fabrication. With high-intensity blue light, however, we encountered material instability, which resulted in fractions of light leaking from undesired emitters, thus requiring recalibration. We managed this issue by limiting the power of blue light. Therefore, in experiments requiring high light intensity and precise spatial addressing, we used red light.

The base of the probe integrates the fiber block, the photonics and the CMOS recording circuits, which are mounted on a printed circuit board (PCB) (Fig. 1d). To accommodate the fiber block and the photonics circuitry, we extended the 5-mm probe base integrating the recording circuits with two wings of 2 and 3 mm. The probe transmits data through a flex cable to a headstage PCB, which connects to a digital data cable.

The data cable and the two-channel optical fiber cable are connected to two modules in a PXI base station, one for digital data processing and one containing blue and red lasers (Fig. 1e). Data acquisition and emitter selection are controlled by one of two widely used open-source software packages, SpikeGLX and the Open Ephys GUI, which were updated for the purpose.

### Electrical and optical characterization

**Extended Data Fig. 3:**
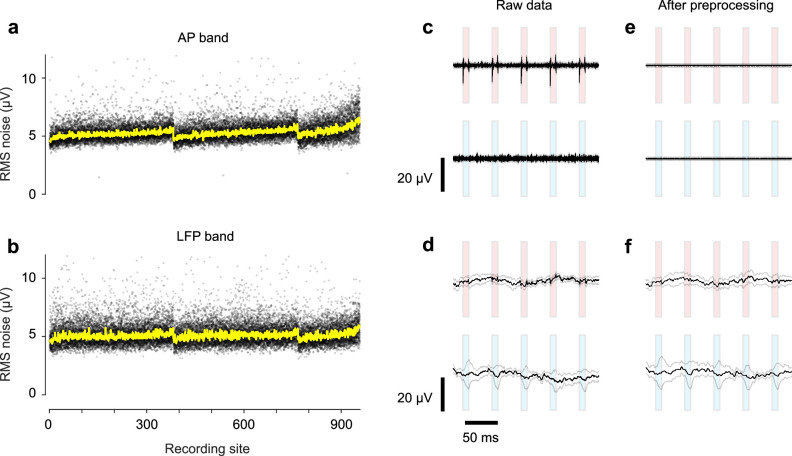
Electrical characterization and light artifact removal. **a**. Input-referred RMS noise levels, measured in saline for 957 recording sites from *N* = 21 probes, in the AP band. Each dot represents a measurement for one site for one probe. Yellow line indicates the median across all probes. **b**. Same, for the LFP band. **c**. Measurements in brain tissue during light activation, showing light artifact for 10 ms red light pulses at 100 μW in the AP band (average of 30 trials, 384 recording sites). The red and blue bars indicate periods of light activation (20 Hz train of 10 ms light pulses). **d**. Same, for the LFP band. **e**. Because the artifact seen for red pulses is highly uniform across recording sites, it is removed by phase shifting and median subtraction of the AP band data. **f**. In the LFP band, the small transients at the onset and offset of each pulse of red light are removed by applying a bandpass filter between 0.1 and 300 Hz.

Despite the addition of the photonics, the electrical performance of the Neuropixels Opto probe remained similar to the widely used Neuropixels 1.0 probes^1^. The average electrode impedance was 138 ± 27 kΩ and the average root-mean-square noise in the AP and LFP bands was 5.45 ± 0.02 μV and 5.33 ± 0.03 μV (mean ± s.e. (standard error), *n* = 20,097 based on 957 sites from 21 probes; Extended Data Fig. 3a,b). These values are below the typical measurements in Neuropixels 1.0 (5.5 μV in AP band and 8.0 μV in LFP band^1^) and Neuropixels 2.0 (7.2 μV for the combined AP–LFP band^2^). This low noise, combined with high site density (100 sites per mm), enables recordings with similar quality to those routinely obtained with established Neuropixels probes.

**Fig. 2:**
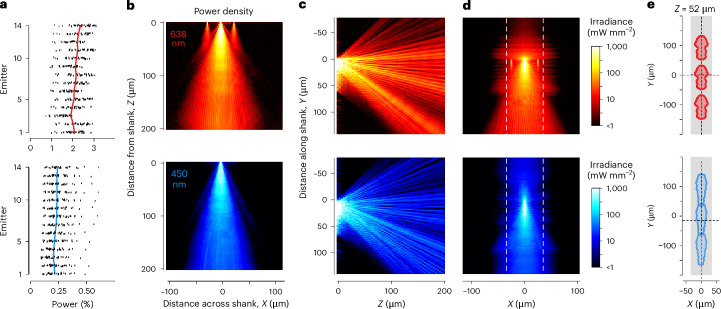
Optical characterization of the Neuropixels Opto probe. **a**, Efficiency of the emitters, showing output light power as a percentage of input power for 14 red (top) and blue (bottom) emitters from *n* = 31 probes. Each dot corresponds to one emitter from one probe. Curves show the average over probes. **b**, Top view, showing light propagation from a red and blue emitter, measured in water. The color (color scale in **d**) indicates the maximum projection. These measurements were made on a test structure where emitters were placed 25 μm apart rather than the 100 μm of the prototype probe and this led to small imaging artifacts visible in the red emission (top), where two emitters to the side of the central one also appear to emit light. **c**, Same data, projected over a side view. **d**, Same data, projected over a front view. Dashed lines delineate the width of the probe. **e**, Section on a plane located 52 μm away from the shank, showing areas where power density is >10 mW mm^−2^ (for a 100-μW output) for three nearby emitters.

As expected, the light at the emitters was a small fraction of the light delivered by the optical fibers; the average emitted power was 2.07% ± 0.02% of the input power for red light and 0.24% ± 0.01% for blue light (mean ± s.e., *n* = 434 based on 14 emitters from 31 probes; Fig. 2a). The attenuation (–16.99 ± 0.06 dB for red, –26.47 ± 0.08 dB for blue) is caused by the coupling of fiber to waveguide (~6 dB), the switching tree (~4 dB), the emitter (simulated at <4 dB) and waveguide propagation, where losses are particularly strong with blue light (3.0 dB cm^−1^, compared to 0.5 dB cm for red light). Because of these losses, achieving 100 μW of output at the emitters requires input powers of ~5 mW for the red light and ~40 mW for the blue light. These powers are easily delivered by external lasers.

The emitted light is sufficient for optogenetic manipulations of neurons in the vicinity of the probe. For ChR2, estimates of the light intensity required for optogenetic stimulation range from 10 mW mm^−2^ (ref. ^40^) to 5 mW mm^−2^ (ref. ^45^) to 1 mW mm^−2^ (ref. ^46^). The variability perhaps reflects differences in preparations, opsin expression levels and distributions and spatial overlap of excitation light with the neuron’s membrane. To be conservative, we used the higher estimates and measured the volume where light power density is at least 10 mW mm^−2^. For a 100-μW output, this volume exceeds 470,000 μm^3^, extending >100 μm from the shank over a wide angular range (Fig. 2b–d and Supplementary Video 1). This is more than sufficient to stimulate neurons in the vicinity of the recording sites and beyond the ~50-μm limit for single-unit recordings^47,48^. The volume where light power density is >1 mW mm^−2^ is considerably larger and exceeds the range of the microscope used for these measurements. Moreover, in a scattering medium such as the brain, this volume is expected to be more homogeneous than in water. We confirmed this expectation using an optical phantom matched to the scattering observed in rodent gray matter (Extended Data Fig. 2b–e).

The close spacing of the emitters means that there is a minimal gap between the patterns of light emitted by different emitters: when slicing the emission profile close to the shank (in a plane ~50 μm away), the area with a power density >10 mW mm^−2^ largely tiles the shank axis (Fig. 2e).

By delivering light away from the recording sites, Neuropixels Opto probes largely avoid photoelectric artifacts. Direct illumination of Neuropixels recording sites with sharp-onset surface stimulation creates a large photoelectric artifact often exceeding 1 mV^1^. By contrast, illumination by Neuropixels Opto emitters, which point light away from the recording sites, caused only a small electrical artifact of ~30 μV. This artifact was present only with red light and only with sharp onsets. It was uniform across recording sites and, thus, easily corrected with standard preprocessing steps (Extended Data Fig. 3c–f).

### Activating local neural populations

**Fig. 3:**
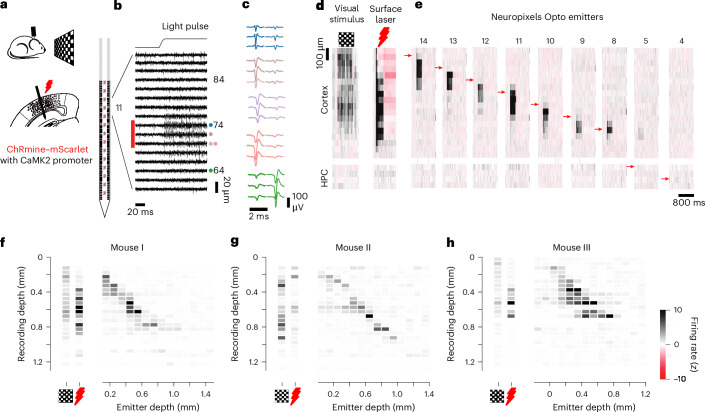
Using Neuropixels Opto to record and activate local neural populations. **a**, We inserted a Neuropixels Opto probe ~1.4 mm deep in the visual cortex of mice expressing the red-sensitive opsin ChRmine (conjugated with mScarlet) in cortical neurons under the CaMK2 promoter. Mice viewed a visual stimulus and an additional red laser illuminated the surface of the posterior cortex. **b**, Simultaneous Neuropixels Opto recordings and optical stimulation with an example emitter (11), obtained with no visual stimulus (gray screen) and no surface laser. **c**, Average spike waveforms from five example single units recorded on sites near emitter 11. The mean waveform was calculated across 100 spikes. **d**, Average firing rate (bin size 50 µm × 2 ms, 40 trials) for the same recording session, plotted as a function of depth, in response to visual stimulation and surface illumination. The color scale bar is shown to the bottom right. **e**, Responses of the same neurons to single emitter activations at different depths (arrows). **f**, Summary of these data showing the average over time of response during stimulation with visual stimulus, surface laser and single emitters (abscissa) at different cortical depths (ordinate). **g**,**h**, Same format, but for example insertions in two other mice. In different insertions, the emitters were at different cortical depths. In **h**, the top emitter was outside the cortex (negative depth), where it elicited no activity. Additional measurements in these mice are shown in Extended Data Fig. 5.

**Extended Data Fig. 4:**
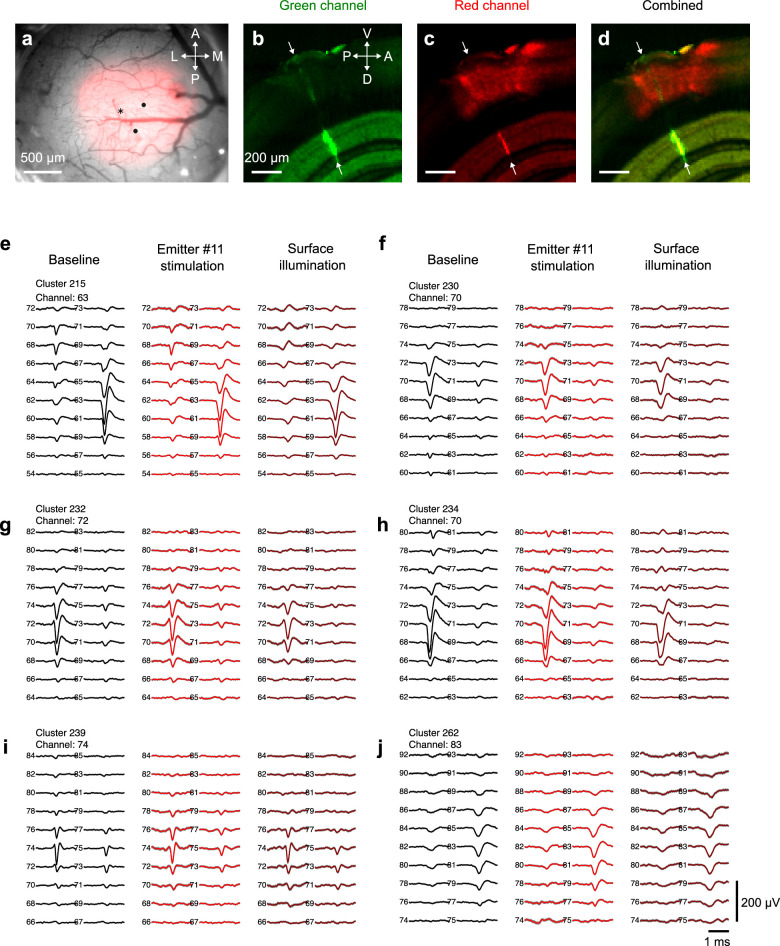
Activating local populations: opsin expression and light responses. **a**. Widefield image of the visual cortex in Mouse I (see Fig. 3), showing the pattern of blood vessels (*gray*), the expression of ChRmine-mScarlet (*red*) and the location of the 3 probe insertions (*black symbols*). The *asterisk* marks the probe insertion shown in the subsequent panels. **b**. Example coronal section obtained with serial section two-photon tomography after perfusion. This image shows the green channel, revealing the track of the Neuropixels Opto probe (*arrows*), which had been immersed in DiO before insertion. The background expression in the hippocampus is due to autofluorescence. **c**. The same coronal section, viewed in the red channel, reveals expression of ChRmine-mScarlet in a patch of cerebral cortex but not in the underlying hippocampus. The DiO marking the probe track results in a faint additional red signal, particularly visible in the hippocampus (*arrows*). **d**. Superposition of the previous two images, showing the probe track in green and opsin expression in red. **e**. Example spike waveform from a unit that passed quality control, showing the spike shape recorded at 10 × 2 sites at baseline (1 s before emitter onset, *left*), during 400 ms emitter stimulation (*middle*), and during 400 ms external surface illumination (*right*). Each trace is the average of 100 spike waveforms. Shaded area (hardly visible) shows ± 1 s.e. **f**-**j**. Five more units stimulated by the same emitter (#11). These units appear in Fig. 3b, c.

We next established the ability of the probes to activate spatially separated neuronal populations. We inserted Neuropixels Opto probes acutely in the primary visual cortex of awake, head-fixed mice following local viral expression of the red-sensitive depolarizing opsin ChRmine^38^ under the CaMK2 promoter (Fig. 3a and Extended Data Fig. 4a–d), which preferentially^49^ targets excitatory neurons. Tapered pulses of red light (638 nm) lasting 400 ms at one example emitter elicited neural activity that was restricted to recording sites near the emitter (Fig. 3b). These recordings had similar quality to standard Neuropixels probes^1,2^; they yielded 0.23 ± 0.09 units per site (median ± m.a.d. (median absolute deviation), *n* = 13 recordings in three mice), similar to the yield obtained in the same area in a set of recordings^50^ with Neuropixels 1.0 probes (0.22 ± 0.10 units per site, median ± m.a.d., *n* = 20 recordings in 20 mice in ten labs, selected with the same quality metrics as our study). We could then readily spike-sort them to obtain the spikes of individual neurons (Fig. 3c), which were unaffected by optical stimulation (Extended Data Fig. 4e–j).

To establish baseline measurements, we presented a visual stimulus (full-field checkerboard) and illuminated the surface of the posterior cortex with a red laser (638 nm, 5 mW). These baseline measurements indicated the presence of recordable neurons throughout the depth of the cortex. We summarize their activity in terms of firing rate as a function of cortical depth (Fig. 3d).

By activating one emitter at a time, Neuropixels Opto probes spatially addressed different subpopulations of these neurons with high resolution (Fig. 3e). Trials with stimulation from different emitters were randomized and randomly interleaved with the baseline trials (visual stimulus or surface laser). Stimulation by single emitters activated small groups of neurons at nearby depths. Taking the average over time of these responses revealed an approximately diagonal matrix (Fig. 3f), reflecting concordance between the location of stimulation and the location of neurons with increased firing rates.

**Extended Data Fig. 5:**
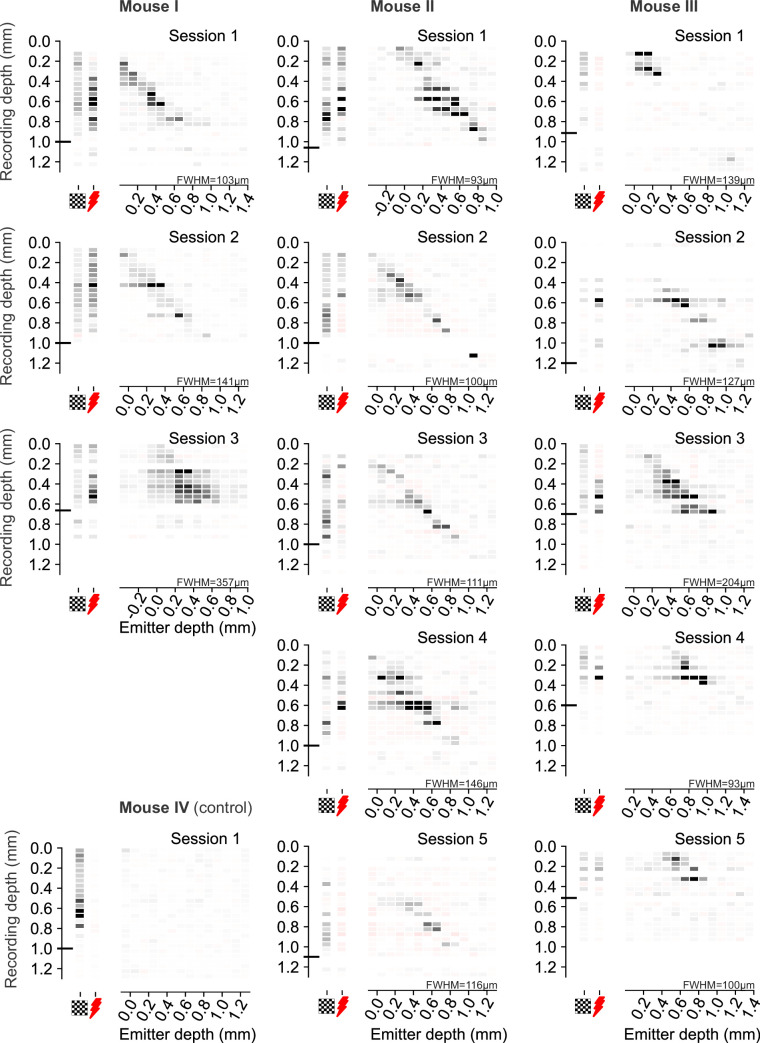
Activating local neural populations: additional examples. Results of all the sessions with the experiments shown in Fig. 3. Format is as in Fig. 3f, showing average over time of response during stimulation with visual stimulus, surface laser, and single emitters (*abscissa*), at different cortical depths (*ordinate*). The horizontal line indicates the putative cortical border. In different insertions the top emitter was at different cortical depths, resulting in different ranges for the abscissa. Mouse IV is a control mouse that did not express any opsin. Accordingly, light stimuli (external or via the emitters) did not elicit any activity.

Similar results were obtained in two other mice (Fig. 3g,h) and across multiple experiments (13 probe insertions in three mice; Extended Data Fig. 5). At the light intensities provided in these experiments, the population of activated neurons extended vertically over 151 ± 71 μm (full-width at half-maximum (FWHM), mean ± s.d., *n* = 13 insertions). This extent represents a lower bound because the recorded neurons are likely to be a fraction of the activated ones. It is comparable to the 100-µm spacing between emitters, suggesting that the probe can provide spatial coverage across emitters. Measurements of LFP and current source density (CSD) confirmed that the emitters generated localized current sources and sinks (Supplementary Fig. 1).

To exclude possible artifacts of light stimulation arising from thermal effects^51^ or retinal activation^52^, we performed two sets of measurements. First, we confirmed that the emitters did not evoke activity in a region that did not express the opsin—the hippocampus (Fig. 3e–h). Second, we ran a control experiment in a mouse that received no virus injection and found no effect of light stimulation (Supplementary Fig. 2).

These results indicate that Neuropixels Opto probes provide concurrent large-scale recordings and fine spatially addressed optogenetics across the depth of a brain structure.

### Driving local circuits

**Extended Data Fig. 6:**
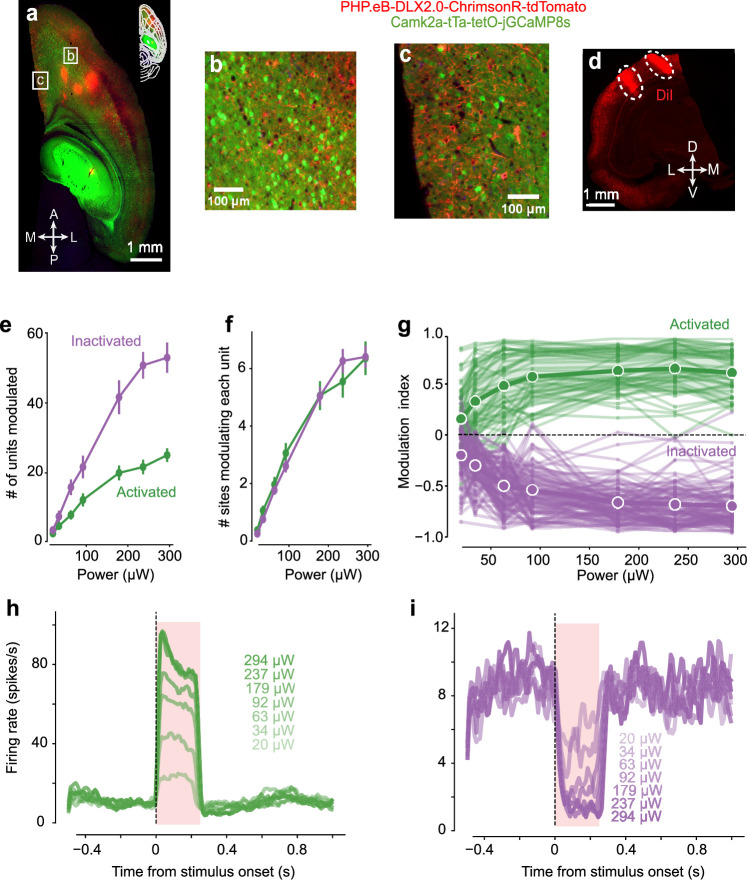
Driving local circuits: opsin expression and power dependence. **a**. Horizontal section through the cleared brain of mouse 2 (Fig. 4), showing fluorescence of ChrimsonR-tdTomato (*red*), which was targeted at inhibitory neurons via a DLX2.0 enhancer, and of GCaMP (*green*), which was targeted at excitatory neurons via a CaMK2a driver. **b-c**, Magnified regions in **a** demonstrate that tdTomato and GCAMP express in distinct neural populations. **d**. Coronal slice of the red channel showing fluorescence of ChrimsonR-tdTomato and of the fluorescent dye DiI (*dashed*), which was used to track probe trajectories. The two probe tracks traverse visual and retrosplenial cortex. **e**. Number of units modulated by light (same criteria as in Fig. 4) as a function of power of light emitted from the probe. Data in this panel and following panels are from a single session in mouse 3. **f**. Number of sites activating each unit at each level of light power. **g**. Modulation index as a function of laser power. All units that are eventually modulated at any power are included in the plot. **h**. Mean PSTH of activated units as a function of laser power. **i**. Same, for inactivated units.

We next tested the ability of Neuropixels Opto probes to drive local circuit effects such as those mediated by synaptic inhibition. We expressed the red-sensitive depolarizing opsin ChrimsonR^37^ in putative inhibitory forebrain neurons by systemic injection^53^ of a DLX2.0 enhancer virus^54^ (Extended Data Fig. 6a–d). We then inserted Neuropixels Opto probes in the mouse dorsal cortex and delivered 250-ms pulses of light at random times from random emitters.

**Fig. 4:**
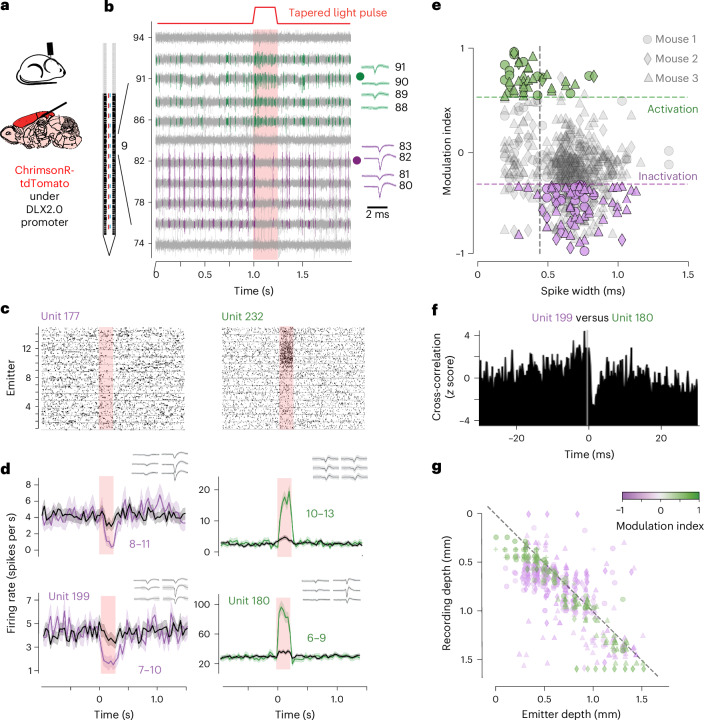
Using Neuropixels Opto to drive local circuits. **a**, We inserted a Neuropixels Opto probe in the dorsal cortex of mice expressing red-sensitive depolarizing opsin ChrimsonR–tdTomato in putative inhibitory neurons (using a DLX2.0 enhancer virus). **b**, Left: electrical signals at 20 recording sites during red-light stimulation from emitter 9. Colors indicate the spikes of two nearby units, one activated (unit 189; green) and one inactivated (unit 208; purple) by light. Right: waveforms across peak channels confirm neural activity. In this panel and subsequent ones, the shaded rectangle indicates the time of optical activation. **c**, Spike rasters for a pair of example units, one inactivated (unit 177) and one activated (unit 232) by light, ordered by the stimulating emitter (ordinate). **d**, Average firing rate (bin size: 40 ms) relative to light onset for those units and for two additional units (199 and 180). Shading indicates ±1 s.e. Insets: waveforms show spike shapes across six peak recordings sites. **e**, Spike width (trough to peak) of average waveforms versus effect of light stimulation, measured by a modulation index (*R*_1_ − *R*_0_)/(*R*_1_ + *R*_0_), where *R*_0_ and *R*_1_ are firing rates before and during stimulus, showing significantly activated units (green) and inactivated units (purple) at *P* < 0.005 (paired two-sided *t*-test). Narrow spikes were defined as width <0.4 ms (vertical line). **f**, CCG between units 199 and 180. **g**, Recording versus emitter depth for significantly modulated neurons. Each neuron appears at one recording depth and at one or more emitter depths (if modulated by light from multiple emitters).

**Extended Data Fig. 7:**
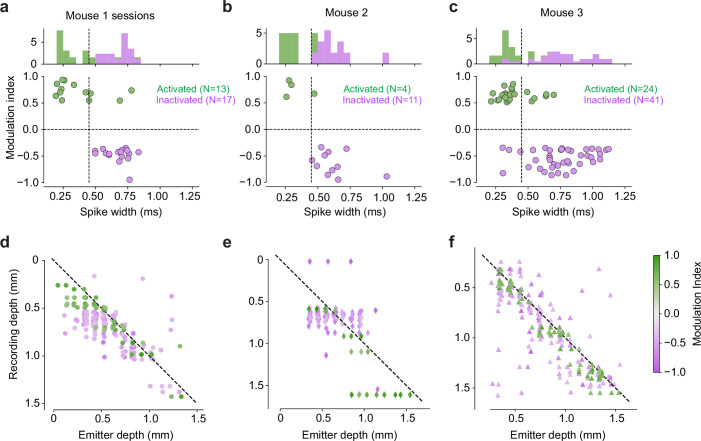
Activation of putative inhibitory neurons in individual mice. **a-c**. Same data as in Fig. 4e, broken down by mouse, showing modulation index (R_1_-R_0_)/(R_1_ + R_0_) (*ordinate*) as a function of trough-to-peak spike width of average waveforms. **d-f**. Same data as in Fig. 4g, broken down by mouse. Each panel shows the recording depth of the neurons as a function of the depth of the effective emitters. Colors indicate modulation index. Each neuron appears at one recording depth and at one or more emitter depths (if modulated by light from multiple emitters).

The results were consistent with localized optogenetic activation of inhibitory neurons and consequent synaptic inhibition of excitatory neurons. Light delivery activated some neurons and inactivated others (Fig. 4a–d). Because the opsin depolarizes neurons in which it is expressed, the activated neurons should correspond to the putative inhibitory neurons expressing the opsin, whereas inactivated neurons should only reflect neurons receiving synaptic inhibition from the activated population. To test this interpretation, we analyzed the spike waveforms^55^ and distinguished putative fast-spiking neurons, which have narrow spikes, from the rest of the neurons, which are likely to be mostly pyramidal and have broader spikes^56^. As expected^55^, the activated neurons were predominantly fast-spiking, whereas the putative pyramidal neurons were predominantly inactivated (Fig. 4e). Moreover, cross-correlograms (CCGs)^56,57^ between some pairs of activated and inactivated neurons were consistent with putative monosynaptic inhibitory connections (Fig. 4f). Both activated and inactivated neurons were observed primarily at depths near the emitter (Fig. 4g), indicating that the localized activation of putative inhibitory neurons engaged local circuit effects. These results were replicated in nine sessions in three mice, providing consistent results (Fig. 4e,g and Extended Data Fig. 7). Both the activation of putative inhibitory neurons and the resulting inactivation of putative excitatory neurons grew in strength with increasing light intensity, especially at low intensities (Extended Data Fig. 6e–i). Taken together, these results confirm that Neuropixels Opto probes are suitable for causing spatially localized circuit effects mediated by synaptic transmission.

### Optotagging nearby neurons

**Extended Data Fig. 8:**
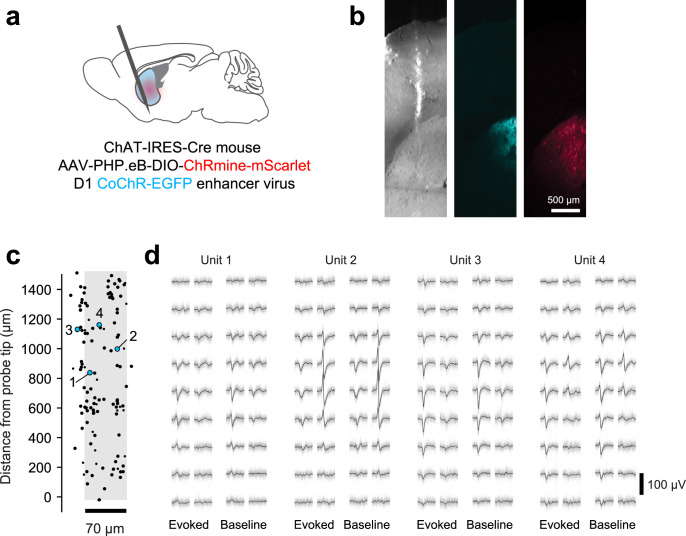
Optotagging with Neuropixels Opto probes preserves spike waveforms. **a**. Schematic of reagents used in an example experiment. **b**. Virtual slice through light sheet volume of the brains used for this experiment, showing the location of the probe track and the regions of blue- or red-shifted opsin expression. **c**. Summary of unit locations within 100 µm of an emitter. Blue-tagged units appear as blue dots; there were no red-tagged units in this experiment. Large dots indicate units that pass quality metric thresholds for the complete session (ISI violations ratio < 0.5, amplitude cutoff < 0.1, presence ratio > 0.8). Small dots indicate units that pass the ISI violations ratio threshold only for the pre-stimulus baseline interval. Numbers indicate location of example units shown in panel **d**. **d**. Example waveforms extracted for each of four units. Each plot shows 50 randomly selected waveforms and their median (*black*), measured during the 10 ms light pulses (‘Evoked’) and in the interval between trials, when no light stimulus was present (‘Baseline’).

When light-sensitive opsins are expressed in a cell-type-specific manner, these cells can be identified in extracellular recordings by optotagging^3–5^, that is, on the basis of their low-latency responses to pulses of light. An ideal tool for optotagging would have minimal artifacts in response to light pulses, minimal activation of neurons outside the range of recording and the ability to deliver both blue and red light wavelengths deep inside the brain. Neuropixels Opto probes meet all of these criteria. Moreover, as demonstrated, by directing the light away from the recording sites, the probes largely avoid photoelectric artifacts (Extended Data Fig. 3c–f). Indeed, the spike waveforms occurring during light stimulation had a similar shape to those occurring when no stimulation was present (Extended Data Fig. 8).

To test the efficacy of Neuropixels Opto for optotagging two cell types in parallel, we recorded in mice expressing a blue-sensitive opsin in one population and a red-sensitive opsin in a second population. Neurons expressing blue-sensitive opsins, such as CoChR^37^, will only be depolarized by blue light, because the sensitivity curves of these opsins drop sharply at longer wavelengths, making them largely unresponsive to red light. By contrast, neurons expressing red-sensitive opsins, such as ChRmine^38^, will be depolarized by both blue and red light, because of the long tail of sensitivity of these opsins to shorter wavelengths. These distinct response patterns make it possible to identify two cell types in the same experiment.

**Extended Data Fig. 9:**
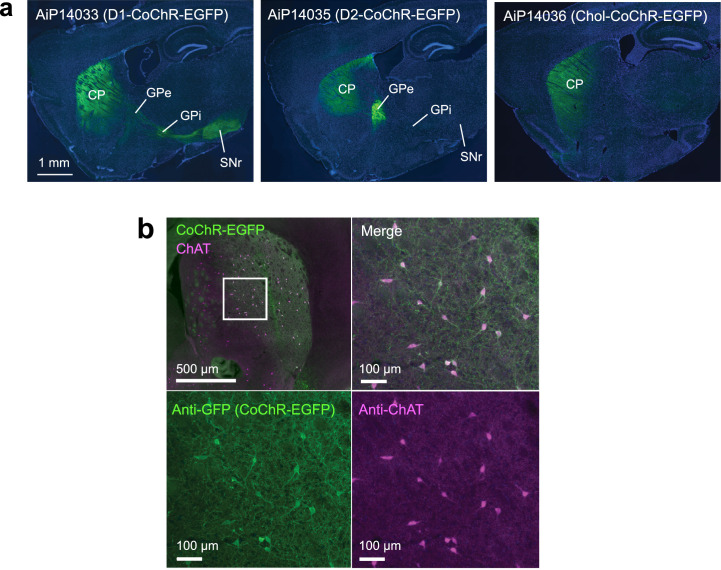
Specificity of CoChR expression in dorsal striatum. **a**, Epifluorescence image of native CoChR-EGFP expression (green) with DAPI counterstain (blue) for D1-CoChR virus AiP14033 (*left*), D2-CoChR virus AiP14035 (*middle*), and Chol-CoChR virus AiP14036 (*right*). Each virus was injected at 2E+9vg dose into adult C57 mice. Abbreviations: CP-caudoputamen, GPe-Globus pallidus externa, GPi-Globus pallidus interna, SNr-substantia nigra pars reticulata. **b**, High colocalization of anti-GFP (reporting CoChR-EGFP fusion protein) and anti-ChAT labeling in the dorsal striatum brain sections from AiP14036 virus-injected mouse.

The red-shifted opsins were expressed with conventional Cre driver lines and Cre-dependent adeno-associated viruses (AAVs), whereas the blue-shifted opsin (CoChR) was expressed with enhancer AAVs^58^. We injected one of three such enhancer AAVs in the striatum to express CoChR–EGFP in direct-pathway (D1) medium spiny neurons (MSNs), indirect-pathway (D2) MSNs or cholinergic (Chol) interneurons (Extended Data Fig. 9). Ex vivo slice recordings revealed peak photocurrents of up to 6 nA in CoChR–EGFP^+^ cells, with mean peak photocurrents of 3.2 nA for D1 neurons, 3.0 nA for D2 neurons and 3.3 nA for Chol neurons (Supplementary Fig. 3). These currents were significantly higher than obtained in D1 neurons expressing ChR2-H134R (ref. ^59^), which had a mean peak photocurrent of 0.4 nA (*P* < 0.001; Welch’s one-way analysis of variance with post hoc *t*-tests corrected for multiple comparisons). Thus, in the experiments in vivo, we chose CoChR because of its higher blue-light sensitivity and higher photocurrents. The red-shifted opsins ChrimsonR and ChRmine were selected for similar reasons.

To optotag neurons, we delivered sequences of 10-ms light pulses^4,5^ at 20 Hz. Previous work has shown that low-latency responses to light pulses in quick succession are only seen in neurons that are activated directly by light (because they are not abolished by a synaptic blocker^5^). Thus, we considered neurons to be optotagged if they satisfied three criteria: (1) they displayed an increase in firing rate compared to baseline in response to a minimum of four of five 10-ms pulses from at least one emitter; (2) the driving emitter evoked at least one spike in 30% of the trials; and (3) the latency of the evoked spikes was <8 ms.

**Fig. 5:**
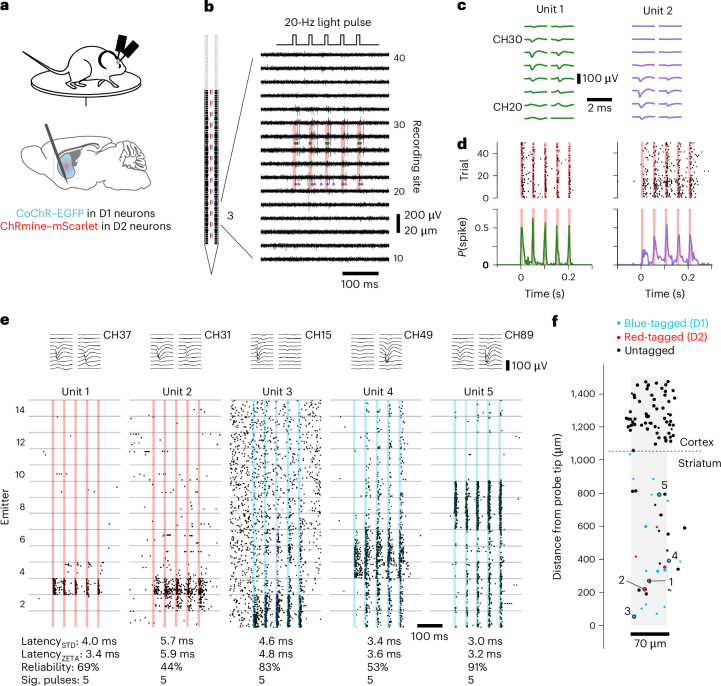
Using Neuropixels Opto for optotagging. **a**, We inserted two Neuropixels Opto probes in the striatum of Adora2a-Cre mice expressing the blue-sensitive opsin CoChR in D1 MSNs (using a D1-MSN-specific enhancer virus) and the red-sensitive opsin ChRmine in D2 MSNs (using a Cre-dependent AAV). Mice were free to run on a disc. After 20 min of recording, we ran the optotagging protocol (10-ms, 20-Hz, 100-µW pulses from each of 14 blue or red emitters, randomly interleaved). **b**, Recorded traces for one trial of light presentation, showing spike times (green and red dots) of two example units tagged by red light from emitter 3. Red shaded areas indicate the timing of the light pulses. **c**, Mean waveforms for the two units highlighted in **b**. **d**, Spike raster and peristimulus time histogram for 50 trials of stimulation from emitter 3, showing a consistent, low-latency response to each light pulse. **e**, Stacked rasters across 50 trials from all 14 emitters for five example units (including the two units from **b–d**). Red and blue shaded regions indicate the time of light presentation. Mean waveforms are shown above each raster. Key optotagging metrics are listed below each raster; units are considered tagged if they have a median latency below 8 ms, a reliability above 30% and a significant (Sig.) response to at least four pulses. Latencies are shown for comparison. **f**, Estimated location of all units passing quality control from a single recording, with units activated by blue or red light shown in blue and units activated by red light only shown in red. Large dots indicate units that pass quality metric thresholds for the complete session (ISI violations ratio <0.5, amplitude cutoff <0.1, presence ratio >0.8). Small dots indicate units that pass the ISI violations ratio threshold only for the prestimulus baseline interval (typical of units with low spontaneous firing rates that are strongly driven by light presentation).

To illustrate this approach, consider an example experiment where we expressed the blue-sensitive opsin CoChR–EGFP^37^ in D1 MSNs and the red-sensitive opsin ChRmine–mScarlet^38^ in D2 MSNs (Fig. 5a). Because the photoartifacts were small and disappeared after preprocessing (Extended Data Fig. 3c–f), spikes were readily identifiable in the raw traces around each light pulse (Fig. 5b). For example, consider two units tagged by red-light pulses from emitter 3 (Fig. 5c). Each unit shows consistent, low-latency spiking responses to each of five 100-µW pulses across 50 trials (Fig. 5d). Spike rasters arranged as a function of emitter location reveal that the strongest responses were evoked by the emitter near the estimated position of the soma (Fig. 5e). For units tagged with blue light, some longer-latency spikes were also evoked by emitters distant from the soma, perhaps because of the small amounts of light leakage mentioned earlier (which are specific to blue light and would require recalibration to be removed). In this recording, we were able to tag the majority of the recorded units in striatum (25 of 39; Fig. 5f), allowing direct comparison of the activity of populations of two cell types in a single structure.

**Extended Data Fig. 10:**
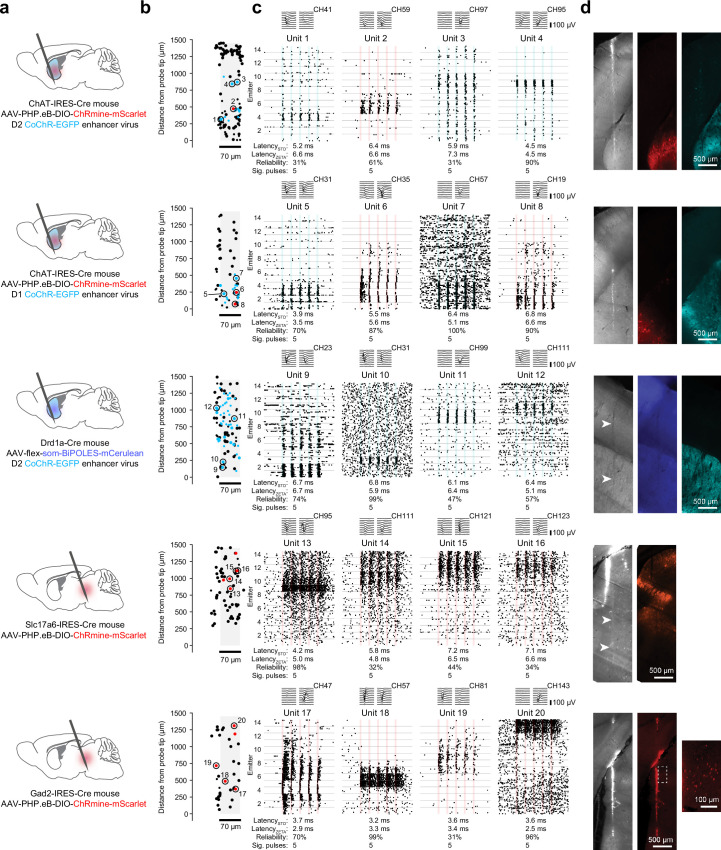
Additional examples of optotagged units. **a**. Schematic of reagents used in five different experiments. **b**. Summary of unit locations within 100 μm of an emitter, showing red-tagged units (*red dots*), blue-tagged units (*blue dots*), and untagged units (*black*). Large dots indicate units that pass quality metric thresholds for the complete session, while small dots indicate units that pass the ISI violations ratio threshold only for the pre-stimulus baseline interval. Numbers indicate location of example units shown in panel **c**. **c**. Stacked raster plots aligned to 20 Hz laser pulses delivered from 14 emitters, for four example units from each session. Colored vertical bars indicate the time of each laser pulse, as well as the opsin (blue- or red-shifted) that this unit expresses. If units responded to both blue and red laser presentations, they were considered to be expressing a red-shifted opsin. **d**. Virtual slices through light sheet volumes of the brains used for the same optotagging experiments shown in panels **a-c**, showing the location of the probe track and the regions of blue- or red-shifted opsin expression. Image colors are based on the colors shown in the schematics in panel **a**. White arrows indicate the probe track in cases where it is faint. In the bottom row, the expanded region shows examples of fluorescent cell bodies that are difficult to see in the zoom-out view.

Similar results were obtained in multiple other experiments where we used a variety of expression strategies for parallel optotagging of D1 and D2 MSNs (as in the example above); D2 MSNs and Chol interneurons; D1 MSNs and Chol interneurons; or glutamatergic or GABAergic neurons in the midbrain (Extended Data Fig. 10). Overall, we optotagged 261 units with Neuropixels Opto across 40 sessions in 26 mice, using CoChR^37^ and ChRmine^38^, ChrimsonR^37^, rsChRmine^60^ or somBiPOLES^61^ (Supplementary Table 1).

**Fig. 6:**
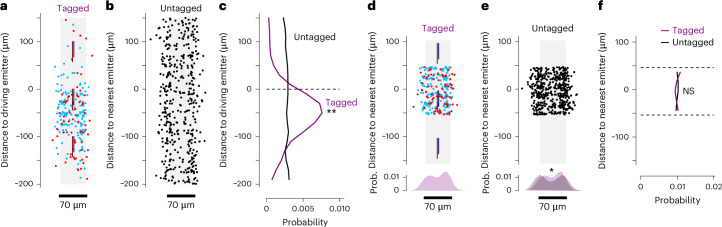
Spatial distribution of optotagged units. **a**, Estimated location of units relative to the driving emitter for units that were optotagged by red light (red dots; *n* = 83 units from 25 sessions) and blue light (blue dots; *n* = 178 units from 26 sessions). **b**, Same as **a**, but for units from the same sessions that were not optotagged (black dots; *n* = 500 randomly selected units from 40 sessions). For these untagged units, the driving emitter is selected arbitrarily. **c**, Probability of tagging a unit as a function of distance from the driving emitter along the shank insertion axis. Most tagged units are located below the driving emitter (deeper in the brain). **P < 1 × 10^−10^ (Wilcoxon two-sided signed-rank test for difference from zero). **d**–**f**, Same data as **a–c**, but plotted relative to the nearest emitter (rather than the driving emitter), showing the probability of being optotagged at any location within 50 μm of an emitter. Bottom: histograms in **d**,**e** show the distributions of unit locations orthogonal to the shank insertion axis. *P < 0.01; not significant (NS), *P* > 0.05 (Kolmogorov–Smirnov test for equality of distributions). Information about cell types and opsins in these experiments is provided in Supplementary Table 1.

We combined the results of these experiments to assess whether optotagging was equally possible at all distances from an emitter or whether there were gaps in coverage. We estimated the two-dimensional location of every unit on the basis of the spatial distribution of its spike waveform and compared the locations of optotagged and untagged units (Fig. 6a,b). We first expressed each unit’s position relative to the driving emitter (the emitter evoking the largest response) and found that optotagged units tended to be located below the driving emitter (closer to the probe tip) (Fig. 6c; *P* < 1 × 10^−10^, Wilcoxon signed-rank test). This arrangement is consistent with the illumination profile of the emitters, which direct light downward along the shank’s long axis (Fig. 2e). We then calculated each unit’s position relative to the nearest emitter so that we could inspect the distribution of tagged neurons in the horizontal and vertical dimensions (Fig. 6d). Horizontally, the tagged units spanned the entire 70-µm width of the shank, with a slightly higher density (than untagged units) near the center of the shank (Fig. 6e; *P* = 0.0036, Kolmogorov–Smirnov test). Vertically (along the length of the shank), the distribution of tagged units was instead indistinguishable from that of untagged units (Fig. 6f; *P* = 0.58, Kolmogorov–Smirnov test). Thus, the 100-µm interemitter spacing is sufficiently dense to leave no gaps in optotagging coverage.

## Discussion

Thanks to integrated CMOS and photonics, Neuropixels Opto probes provide a single device for large-scale neural recordings and spatially addressable optogenetics. Our tests demonstrate that these probes deliver fine spatially addressable optogenetics across the depth of a brain structure, while providing high-resolution recordings. This capability is ideal for investigating the circuit organization of the cerebral cortex^7,13–16^ and other brain regions. In addition, Neuropixels Opto probes are well-suited for optotagging^3–5^, making it possible to identify the cell type of the majority of units in regions such as the striatum.

Our measurements in the cerebral cortex demonstrated that Neuropixels Opto probes can elicit highly localized optogenetic activity. This degree of localization is remarkable given that the layers of the cortex are highly interconnected^13^. Indeed, previous studies that activated individual cortical layers optogenetically found enhancement^13,62^ or suppression^13,63,64^ of activity in other layers. The highly localized results obtained with Neuropixels Opto might be because of lower light levels and to the focused range of the probe’s emitters.

Nonetheless, the spatial resolution obtainable with Neuropixels Opto is limited by the fundamental constraints of one-photon optics in a scattering medium and by the morphology of neurons and opsin expression within those neurons. The scattering of light in brain tissue is such that no emitter can fully restrict activation to highly localized volumes. Moreover, the region of activation can be further increased by the morphology of neurons and the distribution of opsin expression in extended neural processes. For example, the activation of neurons tens of micrometers below the emitter is likely a combined result of light scattering and distributed opsin expression. In this respect, future experiments may obtain more localized activations by using opsins that target specific portions of the neurons, such as the soma or the axon initial segment^65^.

Neuropixels Opto probes represent a marked technical achievement, as they are substantially more complex to fabricate than the existing Neuropixels 1.0 and 2.0 probes. This complexity can be measured as the number of processing steps required for layer depositions, patterning of the layers and quality inspection. The prototype Neuropixels Opto probes presented here require ~740 such processing steps, almost twice as many as for the Neuropixels 1.0 and 2.0 probes, which require ~400 processing steps.

As with Neuropixels 1.0 and 2.0 probes^1,2^, our model is to produce the Neuropixels Opto probes in quantity and distribute them at cost to a wide community. Here, we demonstrated prototype probes. Turning these prototype probes into a mass-producible probe requires additional rounds of fabrication and testing. As with the 1.0 and 2.0 probes, during this process, we will seek to further adjust the design in multiple ways. First, we aim to make the blue-light switches and waveguides more robust by putting them in a separate photonic layer. Second, we aim to integrate photodetectors to monitor the power of each emitter, thus providing active feedback and the ability for users to recalibrate the optical switches if any crosstalk is observed across emitters. Third, we aim to simplify the coupling between lasers and probe and reduce the probe’s form factor by upgrading the CMOS backend to the more compact design developed for the 2.0 probes^2^. Fourth, we aim to further increase the number of red and blue emitters.

We anticipate that Neuropixels Opto probes will become an essential tool for combining high-density electrophysiological recordings with local optogenetic activation or inactivation and for cell-type-specific electrophysiology across the brain.

## Methods

### Design and fabrication

Neuropixels Opto probes monolithically integrate a CMOS recording platform with a dual-color photonic platform^44^.

The integrated circuit (IC) for recording is unchanged from Neuropixels 1.0: a 130-nm silicon-on-insulator CMOS aluminum process with six metal layers. The probe outline and profile are defined with micromachining. The TiN recording sites (12 × 12 µm^2^) are arranged in a 2 × 480 linear array, spaced 20 µm vertically and 48 µm horizontally.

The photonic layer is integrated between the IC and the recording electrodes (Extended Data Fig. 1). Plasma-enhanced chemical vapor deposition (PECVD) is used to create 150-nm-thick SiN waveguides, allowing single-mode operation at 450 nm (blue) and 638 nm (red) wavelengths. Propagation loss for these waveguides (measured on 84 dies over a full 200-mm wafer) was 2.97 ± 0.10 dB cm^−1^ at 450 nm and 0.52 ± 0.01 dB cm^−1^ at 638 nm. The waveguides are routed through circular bends with a 40-µm radius, with minimal losses of 0.006 ± 0.003 dB (blue) and 0.016 ± 0.001 dB (red) per 90° bend.

To accommodate the photonic switching and the fiber block, we extended the original 5-mm probe base integrating the neural readout circuits with wings measuring 2 mm and 3 mm, respectively.

The fiber block area includes eight grating couplers, aligned to an eight-fiber block with a 127-µm pitch. The two central gratings couple the 450-nm and 638-nm light from the fibers. The remaining six are used for active alignment and for measuring coupling losses. The average coupling loss at 450 nm was 5.5 dB (*n* = 47 probes).

The photonic switching area includes two four-level, current-driven photonic binary switching trees, routing the light to any of the 16 (2^4^) output waveguides (Extended Data Fig. 2a). The switches are Mach–Zehnder interferometers, with phase shifters that operate on the thermo-optic effect. For a 2*π* phase shift, their efficiency is 16 mW for blue and 35 mW for red, with a bandwidth of 20 kHz. To select a specific emitter, four switches must be driven, requiring a total of eight current sources for the two colors. The total insertion loss of the switching tree is 4.3 dB for blue and 3.5 dB for red.

The 2 × 14 emitters are spaced 100 µm apart, covering the first 1.5 mm from the tip of the shank. To achieve broad angular emission, we used higher-order, apodized Bragg gratings, which spread the emission over multiple diffraction peaks. This precluded the use of a reflector, resulting in a coupling efficiency of 40%.

We expected the cumulative insertion losses from the fiber to the emitter to be ~25 dB for blue and ~16 dB for red. Measurements from 31 probes confirmed these expectations, with average total loss values of 26.5 dB for blue and 17.0 dB for red.

To integrate the photonics circuitry while maintaining CMOS performance, we addressed two main challenges. First, adding the photonics, particularly the high-stress SiN waveguide layer, could bend the shank excessively. To counteract this bend, we deposited a SiN compensation layer and we used the SiN capping layer for fine-tuning, achieving a tip deflection <±200 nm. Second, to prevent scattered light from the waveguides from interfering with the CMOS circuitry, we implemented a TiN/Al-based light-blocking layer. The oxide cladding layers of the PECVD SiN waveguides were kept as thin as possible to minimize their impact on shank bending and thickness.

### Light source

Except where indicated, the light source was a PXI-mounted laser module emitting at 450 nm and 638 nm (Quantifi Photonics), connected to the Neuropixels Opto probe by an optic fiber.

After the electrical characterization measurements, the light power was equalized across emitters on the basis of the measured loss for each emitter.

Emitters were selected using SpikeGLX or the Open Ephys GUI, which were updated for the purpose. Analog modulation of laser output was achieved by controlling a PXI-mounted National Instruments data acquisition module with a Python or MATLAB script, with network messages used to synchronize light output with emitter selection.

### Electrical and optical characterization

#### Electrical characterization

Measurements were performed in a grounded Faraday cage. The probe shank was immersed in PBS solution. The channels were configured to ×1,000 gain with an external reference connected to the ground pad of the probe. First, we measured gain. We applied a sinusoidal test signal of 500 μV (peak to peak) at 1.5 kHz or 150 Hz (for AP or LFP band) to the PBS solution using a platinum counter electrode. We recorded the probe signal and calculated the gain at the two frequencies. Second, we measured noise. We grounded the PBS solution using the external reference and ground contact pads on the flex cable. We recorded the probe signal and calculated the integrated noise in the frequency bands for AP and LFP. We then divided the noise by the gain to obtain the input-referred noise.

#### Optical characterization

To measure the full probe loss, we inserted the shanks into an integrating sphere (AvaSphere-30-IRRAD, Avantes). To measure the emitter radiation pattern, we used a Nikon Eclipse microscope with a water-immersion objective (Nikon Fluor, ×60, numerical aperture (NA): 1.0) and a motorized three-dimensional (3D) fiber coupling stage (based on the PI Q-545 linear stage). Images were acquired with a scientific CMOS camera (Hamamatsu Orca Flash) providing a field of view of 220 × 220 μm. The field of view was centered on the emitter and the objective was focused on the waveguide plane. A 200-μm *z* scan was performed with a 4-μm step.

These measurements were made on a test structure: a 1-cm-long waveguide with a standard grating coupler on one side and the emitter on the other. Light was coupled into the structure with a horizontally placed 40° angle polished fiber, which was aligned to the standard grating coupler. The test structures were arranged in blocks with many waveguides 25 μm apart. For red light, we could not avoid coupling some light into the neighboring waveguides, resulting in small artifacts where a small amount of light was visible in neighboring emitters (Fig. 2b, top). These artifacts were specific to the test structure layout and were not present at the emitters of fully constructed probes.

### Measurements in an optical phantom

To measure the light projected by a Neuropixels Opto emitter onto individual planes (optical sectioning), we placed the probe at various distances from a fluorescent imaging plane.

The plane was obtained by coating a Superfrost Plus glass slide (631-9483, Menzel, Avantor Sciences) with ~10 µl of carboxyl quantum dots (Qdot 655 ITK, Q21321MP, Thermo Fisher). The Qdots were spread into a thin, even layer by placing a clean glass coverslip at the edge of the droplet and dragging it across the surface to promote capillary action. We then placed the slide in a 45 °C incubator on a shaker for 2–3 min to promote adhesion of the Qdots to the slide and allow the aqueous solvent to partially evaporate. A small drop (~15 μl) of optical adhesive (NOA 81, Norland Products, Thorlabs) was applied over the dried Qdot layer. Again, we used a coverslip to gently spread the adhesive into a thin, uniform film across the slide. We then cured this adhesive layer using ultraviolet (UV) light at 365 nm for ~3 min to ensure complete crosslinking of the polymer. This curing step rendered the Qdot-coated slide watertight and suitable for extended aqueous immersion. The final height of the coating was measured at ~30 µm using a dial gauge with resolution of 10 µm.

A micromanipulator (uMp-4, Sensapex) varied the distance between the probe and the imaging plane and a camera (BFS-U3-50S5M-C, Teledyne FLIR) focused on the imaging plane using an air objective (UPlanFL N, ×4, NA: 0.13, Olympus) acquired images through an optical filter (FF01-676/37-25, Semrock). The resulting resolution was 2.3 µm per pixel. The probe was first immersed in water to establish baseline measurements and then in 1% milk. This medium was chosen because its estimated^66^ reduced scattering coefficient ${\mu }_{s}^{{\prime} }$ (22.5 cm^−1^) is in the range of the values measured in rodent gray matter^66^ (20–30 cm^−1^).

### Spike sorting and quality metrics

In the brain recordings, spike sorting was performed with Kilosort^2,67^, typically using SpikeInterface^68^. For quality control, we selected clusters with interspike-interval (ISI) violation ratio <0.5, amplitude cutoff <0.1 and presence ratio >0.8. These quantities are defined at https://spikeinterface.readthedocs.io/en/0.98.0/modules/qualitymetrics.html.

### Activating local neural populations

The experiments demonstrating recording and activation of local neural populations (Fig. 3 and Extended Data Figs. 4 and 5) were performed according to the UK Animals Scientific Procedures Act (1986) under personal and project licenses released by the Home Office, following review from the Animal Welfare and Ethical Review Body at University College London.

#### Mice and viral strategy

The experiments were performed on four adult mice (aged 10–16 weeks at the time of headplate implantation): two wild-type males (C57BL/6, Charles River) and two double-transgenic females (Ai32 (ref. ^69^) × PV-Cre (ref. ^70^); JAX, 012569 and 008069), which expressed the blue-sensitive opsin ChR2 in inhibitory (PV^+^) neurons. Three of the mice were injected with a virus expressing red-sensitive opsin ChRmine in CaMK2^+^ neurons^38^ (AAV-8-CaMKIIa-ChRmine-mScarlet-Kv2.1-WPRE, GVVC-AAV-194, Stanford Viral Core). Cells expressing CaMK2 are largely^49^ (albeit not uniquely^71–74^) excitatory. An additional wild-type male (C57BL/6, Charles River) with no opsin expression was used as a control.

#### Main surgery

An initial surgery was performed to implant a headplate, perform a craniotomy and inject a virus. Procedures were adapted from an established protocol^75^. Briefly, mice were injected with dexamethasone (intramuscularly) and then anesthetized with isoflurane (3% for induction, 1–1.5% for maintenance). Appropriate hydration and temperature control were provided. A steel headplate was attached to the skull with dental cement (Super-Bond C&B, Sun Medical). The skin margins were attached to the cranium with tissue adhesive (Vetbond, 3M). A 3-mm craniotomy was performed, centered on the left primary visual area (VISp; ~3.7 mm posterior and ~3 mm lateral). A glass pipette (Drummond Scientific), beveled to form a ∼25–40-μm tip (EG-45 Microgrinder, Narishige), was lowered 150, 300 and 550 μm into the brain to deliver 70 nl of viral vector solution (2 × 10^12^ viral genome copies (vg) per ml) at each depth (Nanoject II, Drummond Scientific), with 3-min pauses between depths and a 5-min pause at the bottom. Injections were performed in 4–5 locations in the VISp, 500–750 μm apart. The total volume of virus solution delivered was 840–1,050 nl. The craniotomy was then covered with a removable window^76^ comprising two 3-mm circular cover glasses (#1) attached to a 5-mm circular cover glass (#1, Warner Instruments) using optical adhesive (Norland Optical Adhesive NOA 61, Thorlabs). The remaining exposed cranium and the skin margin were covered with cement (Super-Bond C&B). Postoperative treatment was provided for 3 days with carprofen in drinking water. Later, the mice were handled and habituated to the head-fixed recording rig for 30–60 min for at least 4 days before any recordings.

#### Widefield imaging

We waited 3–4 weeks for the virus to fully express and found the locations of virus expression using epifluorescence widefield imaging involving an illuminator (X-Cite DC200, Excelitas), a trinocular (Nikon C-TF), a ×4 (NA: 0.13) air objective (UPlanFL N, Olympus), filter cubes for GFP and TurboFP635 (Chroma, VT) and a scientific CMOS camera (PCO.Edge 5.5 CLHS, Excelitas). We then prepared a replacement glass window with holes in the appropriate locations.

#### Window replacement

At least 12 h before the first recordings, we performed a brief procedure to replace the glass window with one that had drilled holes. Mice were anesthetized using isoflurane (3% for induction, 1–1.5% for maintenance). The cement around the glass window was removed with a dental drill and the new window was implanted in its place. We made an incision in the dura at the recording site to allow easier probe insertion, then covered the craniotomy with artificial dura^77^ (Duragel, Cambridge NeuroTech) and sealed the holes with Kwik-Cast (WPI).

#### Recordings

A Neuropixels Opto probe with metal dovetail was mounted on a probe holder (designed by Howard Hughes Medical Institute) and then on a four-axis micromanipulator (uMp-4, Sensapex). In some recordings, we labeled the probe tip using Vybrant CM-DiI or DiO (V22888 or V22886, Thermo Fisher). We lowered the probe to the brain surface and inserted it at 2 µm s^−1^, typically reaching a depth of 1.2–1.4 mm. We then waited 15 min for the probe to settle and started the recordings. During recordings, we controlled the probe and acquired signals using SpikeGLX, with standard gain settings of 500× and 250× for the AP and LFP bands. After recordings, we sealed the hole in the glass window with Kwik-Cast and cleaned the shank in distilled water overnight. Occasionally, Duragel or tissue sticking to the shank required rinsing with a 1% Tergazyme solution for 30 min before placement in distilled water.

#### Photostimulation

To activate neurons with light, we presented a 400-ms tapered square pulse of red light (638 nm). To minimize light artifacts^1^, the pulse had a smooth onset given by half a cycle of a 40-Hz sine wave. The pulses were randomized across the 14 emitters, randomly interleaved with control trials (external illumination, visual stimulation and gray screen) and repeated 40 times. The average intertrial interval was 1.0 s. External optical activation was performed using a 638-nm diode laser (LuxX 638-150, Omicron-Laserage Laserproducte) through a 200-mm (NA: 0.22) patch cord (M122L02, Thorlabs), a collimator (F280FC-A, Thorlabs) and a focusing lens (*f* = 50 mm; LA1213-A, Thorlabs) positioned ~5 cm above the brain surface. Visual stimulation (full-field checkerboard, flickering at 4 Hz, 100% contrast, 1 s) was displayed on an LCD screen (LP097Qx1, LG).

#### Data processing

Signals in the AP band were filtered with a 300–12,000-Hz three-pole Butterworth bandpass filter, followed by analog-to-digital converter phase-shift correction and common median subtraction. Sessions were spike-sorted with Kilosort 4 (ref. ^67^) using default parameters. Sorting jobs were generated and executed using custom Python packages (https://github.com/spkware/spks). Quality metrics were calculated by adapting code from SpikeInterface^68^ and neurons were selected on the basis of criteria described above. For multiunit activity, we used less stringent criteria: amplitude cutoff <0.1, presence ratio >0.7 and ISI violations <2.

#### Measurements of yield

To estimate the yield in terms of units per recording site, we counted the number of neurons that passed quality control and were located in the visual cortex. We compared these numbers with those in the IBL database^50^ for recordings (with Neuropixels 1.0 probes) in the same general region (2,200–3,800 anterior–posterior, 1,800–3,800 medial–lateral, maximum depth: 4,100), selected with the same quality control metrics. The median yield with Opto probes (0.23 units per site) resembled that of IBL recordings (0.22 units per site).

#### Local field potential

Neuropixels probes record LFPs sampled at 2.5 kHz. We applied bandpass filtering (5–60 Hz) using noncausal, zero-phase delay filtering. CSD maps were then generated from average LFPs using established methods^78^. LFPs from the uppermost and lowermost channels were duplicated, smoothed across adjacent channels with a weighted filter and the second spatial derivative was calculated using a sampling interval of 40 µm. The resulting CSD data were linearly interpolated.

#### Width of activated region

To quantify the spatial spread of optogenetically evoked activity, we computed mean *z*-scored firing rates for each recording site and stimulating emitter, binned in depth intervals of 50 µm. We then calculated a single activation profile as a function of distance from stimulating emitter, by realigning relative to stimulation depth and averaging across all stimulation emitters in cortex. We fitted this profile with a double-exponential decay function and computed the FWHM from the fitted profile.

#### Histology

Following the recordings, mice were perfused with 4% paraformaldehyde (PFA; 28908, Thermo Fisher). The brain was dissected, postfixed in PFA for 24 h and stored in 10% PBS for at least 48 h. We imaged 3D stacks of the brains in a custom-made serial section two-photon tomography microscope^79^. Images were acquired using ScanImage (Vidrio Technologies) and the hardware was coordinated with BakingTray.

### Driving local circuits

The spatially resolved neural inactivations (Fig. 4 and Extended Data Figs. 6 and 7) were performed in accordance with protocols approved by the Institutional Animal Care and Use Committee (IACUC) at the University of Washington.

#### Mice and viral strategy

The experiments were performed on two adult female mice and one adult male mouse (aged 19 weeks at the time of headplate implantation) with transgenic expression of GCAMP8s in CAMK2^+^ neurons (CaMK2a-tTA.tetO-G8s). Mice were injected with a virus expressing red-sensitive opsin ChrimsonR–tdTomato under the control of the DLX2.0 enhancer^54,80^ (AAV-PHP.eB-DLX2.0-ChrimsonR-tdTomato; Addgene, plasmid 229775). The virus (100 μl, 1.6 × 10^13^ vg per ml) was retro-orbitally injected under anesthesia^81^ (1–4% isoflurane in O_2_) when the mice were 4–6 weeks old.

#### Implant surgery

Implant surgeries were performed after mice were at least 48 days old. Mice were anesthetized with isoflurane (1–4% in O_2_) and subcutaneously administered analgesics carprofen (5 mg kg^−1^) and lidocaine (2 mg kg^−1^). The skin and periosteum were cleared to reveal the dorsal skull. The edges of the implant were secured to the skull with cyanoacrylate (VetBond, World Precision Instruments) to protect the underlying muscle. A 3D-printed recording chamber was implanted on top of the skull using dental cement (Metabond, Parkell). Fast-curing optical adhesive (Norland Optical Adhesive 81, Norland Products) was applied to the surface of the skull and cured with UV light. A titanium headpost (ProtoLabs) was then cemented to the posterior end of the recording chamber. Carprofen (0.05 mg ml^−1^) was given for 2 days in water after surgery. Mice were allowed to recover in the home cage for at least 1 week before habituation and head fixation.

#### Recordings

Over 3 h before a recording session, the mouse was anesthetized (1–4% isoflurane in O_2_) and a 2–3-mm craniotomy was performed over the left visual cortex and right motor cortex. Craniotomies were sealed with transparent Duragel (Dow Corning 3-4680 Silicone Gel). After recovery from anesthesia, mice were head-fixed. The Neuropixels Opto probe was mounted on a micromanipulator and manually driven to the craniotomy at a 45° angle to accommodate an overhead laser. Real-time electrophysiological data were monitored as the probe was driven through into the brain. Insertions aimed to avoid blood vessels. If a probe could not successfully record from a craniotomy, a new surgery was performed at a later date. Probes were driven from the brain’s surface to their final depth at 200 μm min^−1^ and were allowed to settle there for 10 min before recording data with SpikeGLX. We used internal tip referencing with gain settings of 1,000× and 1,000× for the AP and LFP bands. Probes were slowly removed from the brain (~1 mm min^−1^) at the end of recording. Probes were submerged in a 1% Tergazyme solution overnight and then rinsed in deionized water the next day to clean debris off the shank of the probe.

#### Photostimulation

To activate and inactivate neurons with light emitted from the probe, we presented a 250-ms tapered square pulse of red light (638 nm) from a randomly chosen emitter with an average intertrial interval of 1.4 s. To minimize light arteficts^1^, the pulse was tapered, ramping linearly up for the first 25 and then down for the last 25 ms. Experiments in mice 2 and 3 were performed using an Oxxius LBX-638nm diode laser. Calibrations were performed to ensure both lasers supplied a similar amount of light to the probe during experiments.

#### Data processing

The data were acquired with SpikeGLX, preprocessed with SpikeInterface^68^ (decompression, phase shift, high-pass filter and median subtraction), spike-sorted with Kilosort 4 (ref. ^67^) and inspected with Neuropyxels^82^. Light-activated neuron–emitter pairs were defined as light-activated if the firing rate during the 250-ms light stimulus increased by >300% with *P* < 0.05 and as inactivated if it decreased by >50% with *P* < 0.05. The modulation index was computed as (*R*_1_ − *R*_0_)/(*R*_1_ + *R*_0_), where *R*_0_ and *R*_1_ are the firing rates before and during stimulus. When calculating modulation index (Fig. 4e), we averaged the firing rates (*R*_0_ and *R*_1_) for the two most proximal emitters superficial to each unit. CCGs were computed in a ±30-ms window with 0.2-ms bin size (function ‘plot_ccg’ in Neuropyxels^82^). CCGs were normalized by *z* scoring against the mean and s.d. of the outer portions of the histogram (excluding ±6 ms around zero). CCGs were visually inspected to find examples consistent with excitatory and inhibitory interactions (for example, Fig. 4f).

#### Histology

Following completion of recordings, mice were transcardially perfused with PBS (50 ml at 5 ml min^−1^) followed by 4% PFA in PBS. Brains were extracted and postfixed in 4% PFA overnight at 4 °C. Fixed brains were incubated in SHIELD OFF buffer (5 ml of deionized water, 5 ml of SHIELD-Buffer and 10 ml of SHIELD-Epoxy) at 4 °C for 5 days and then transferred to SHIELD ON buffer at 37 °C for 24 h. Samples were cleared in LifeCanvas delipidation buffer at 45 °C with shaking (5 days per hemisphere), washed overnight in PBS and 0.02% sodium azide at 37 °C and incubated in EasyIndex until transparent. Endogenous GCaMP8s and ChrimsonR–tdTomato were imaged by lightsheet microscopy. Samples were mounted in 2% agarose in EasyIndex and *z* stacks were acquired through cortical regions to visualize calcium indicators and GABAergic neuron distributions. More information is available online (https://lifecanvastech.com/beginners-guide-to-tissue-clearing-with-lifecanvas-products/).

### Optotagging nearby neurons (AI)

The subcortical optotagging experiments (Figs. 5 and 6 and Extended Data Figs. 8 and 10) were carried out in accordance with protocols approved by the IACUC at the Allen Institute.

#### Mice and viral strategy

Experiments were performed on 26 adult mice (11 males, 15 females; aged 11–28 weeks at the time of headframe implantation). We used eight transgenic lines:

- Chat-IRES-Cre^83^ (JAX, 031661)
- Chat-IRES-Cre-neo^83^ (JAX, 006410)
- Sst-IRES-Cre (JAX, 028864)
- Drd1a-Cre^84^ (JAX, 037156)
- Adora2a-Cre (MMRRC, 36158)
- Slc17a6-IRES-Cre^85^ (JAX, 028863)
- Ntrk1-IRES-Cre (MMRRC, 15500)
- Gad2-IRES-Cre^86^ (JAX, 028867)

In addition, some mice received one or more of the following viruses injected stereotaxically:

- pAAV-Syn-FLEX-rc(ChrimsonR-tdTomato) (Addgene, 62723), 3.39 × 10^13^ genome copies (GC) per ml
- pAAV-Ef1a-DIO-ChRmine-mScarlet-WPRE (Addgene, 130998), 1.07 × 10^13^ GC per ml
- pAAV-Ef1a-DIO-rsChRmine-oScarlet-Kv2.1-WPRE (Addgene, 183529), 7.60×10^12^ GC per ml
- hSyn-DIO-somBiPOLES-mCerulean (Addgene, 154951), 2.41 × 10^12^ GC per ml
- AiP14033: pAAV-AiE0779m_3xC2-minBG-CoChR-EGFP-WPRE3-BGHpA (Addgene, 214852), 1.41×10^13^ GC per ml
- AiP14035: pAAV-AiE0452h_3xC2-minBG-CoChR-EGFP-WPRE3-BGHpA (Addgene, 214853), 5.15 × 10^13^ GC per ml
- AiP14036: pAAV-AiE0743m_3xC2-minBG-CoChR-EGFP-WPRE3-BGHpA (Addgene, 214854), 4.13×10^13^ GC per ml

The example experiment (Fig. 5) involved Adora2a-Cre mice (MMRRC, 36158) injected with viruses expressing CoChR–EGFP (Addgene, 214852) and ChRmine–mScarlet (Addgene, 130998).

#### Surgery

Mice were anesthetized and placed in a stereotaxic frame. The dorsal scalp was removed, the skull was leveled and the bregma was located using tooling adapted from a previously described headframe and clamping system^87^. An outline of the implant location was etched using a custom tracing tool and the assembled headframe was cemented in place. A craniotomy was performed using the traced implant shape as a guide and the dura was removed. If the mouse was to receive viral injections, these were delivered stereotaxically through the craniotomy. Afterward, the prepared 3D-printed SHIELD artificial skull^88^ covered with silicone was placed in the opening. The edges of the implant were sealed to the skull using a light-curing cyanoacrylate adhesive (Loctite 4305) and reinforced with dental cement. Finally, a removable plastic cap was placed over the well to protect the implant’s silicone coating. After at least 1 week of recovery and before the first recording, the mouse was anesthetized to remove the layer of silicone before inserting a ground wire into the grounding hole in the implant until it rested on the surface of the brain. A Duragel mixture was then poured over the implant to a thickness of >1 mm and then allowed to cure for >24 h (ref. ^89^).

#### Recordings

To allow post hoc identification of probe tracks, probe shanks were coated with DS-DiD (2 mM in ethanol; Thermo Fisher, D12730) by immersing them five times in a well filled with dye. Recordings involved one or two probes (one per hemisphere). Each probe was mounted on a three-axis micromanipulator (New Scale Technologies) on a custom modular insertion system. We used Pinpoint^90^ to select the insertion coordinates and approach angle for the target structure. Probes were manually driven to the appropriate hole in the SHIELD implant and lowered to the brain surface, identified by observing real-time electrophysiological signals. If the insertion needed adjustment (for example, to avoid blood vessels), the probe was retracted out of the Duragel and, if needed, another target hole was selected. Once all probes reached the brain surface, each probe was automatically inserted to its target depth at a speed of 200 μm min^−1^ and allowed to settle for 10 min. Neuropixels data were acquired using the Open Ephys GUI^91^ with gain settings of 500× and 250× for the AP and LFP bands. Videos of the eye, face and/or body were acquired with USB3 cameras (Teledyne FLIR) and Bonsai software^92^. At the end of the recording session, probes were slowly retracted from the brain (~1 mm min^−1^), submerged in a 1% Tergazyme solution overnight and then rinsed in deionized water the next day.

#### Optotagging

To identify recorded units reliably activated by light, we delivered 20-Hz trains of 10-ms laser pulses separated on average by 300 ms. Blue light (450 or 473 nm) or red light (638 nm) was delivered using the PXI-mounted laser module or a two-channel laser combiner (Oxxius). Optotagged units were identified by their significant increase in firing rate to at least four pulses from at least one emitter (*P* < 0.05, Holm–Šidák adjustment for multiple comparisons), a spike latency <8 ms (defined as the time when the spike rate reached 2 s.d. above baseline) and a mean response reliability >0.3 (proportion of trials with at least one spike evoked during the laser presentation). If units were tagged by both blue and red light, they were considered red-tagged; red-shifted opsins can be activated by blue light but blue-shifted opsins are insensitive to red light.

#### Data processing

Neuropixels raw data files were compressed using WavPack^93^ and transferred to an Amazon S3 bucket. We used a custom Nextflow pipeline running on the Code Ocean compute platform to run preprocessing, spike sorting and curation. First, the data were decompressed and denoised using phase shift, high-pass filter and median subtraction. Then, spike sorting was performed using Kilosort 2.5 (ref. ^2^). Units with waveforms unlikely to originate from APs were automatically labeled as ‘noise’ using pretrained models from the UnitRefine toolbox. Lastly, we calculated quality metrics for each unit and applied the quality control criteria mentioned in an earlier section. Each unit’s location along the shank was estimated on the basis of monopolar triangulation using the waveform amplitudes for recording sites within 75 μm of the peak. All these calculations were made with SpikeInterface^68^. In Fig. 5e, latencies were calculated using the ZETA test^94^.

### In vitro characterization of enhancer viruses

Experiments to characterize enhancer virus expression patterns (Extended Data Fig. 9) and evoked photocurrents (Supplementary Fig. 3) were carried out in accordance with protocols approved by the IACUC at the Allen Institute.

#### AAV vector construction

AiP14033 (Addgene, plasmid 214852), AiP14035 (Addgene, plasmid 214853) and AiP14036 (Addgene, plasmid 214854) were created by subcloning the CoChR–EGFP fragment from Addgene plasmid 59070 (a gift from E. Boyden) using restriction enzyme digestion (BamHI/EcoRI) and ligation into AiP13044 (Addgene, plasmid 191706), AiP12982 (Addgene, plasmid 191709) and AiP13038 (Addgene, plasmid 191720). AiP13276 (Addgene, plasmid 214842) was used as a control vector for comparing enhancer-driven ChR2-H134R–EYFP versus enhancer-driven CoChR–EGFP. Plasmid integrity was verified by Sanger DNA sequencing. All AAV plasmids were propagated in NEB stable *Escherichia* *coli* under 30 °C growth conditions to prevent spurious DNA rearrangements.

#### AAV packaging and titer determination

Small-scale crude AAV preps were generated using the HEK293 cell culture and triple transfection method as described elsewhere^95^. Viral preps were purified by iodixanol gradient centrifugation and subjected to droplet digital PCR for titer determination.

#### Stereotaxic injection of AAV vector

Adult C57Bl/6J mice were deeply anesthetized with isoflurane and placed into the stereotaxic injection frame. AAV virus was injected bilaterally into the dorsal striatum (dSTR) using the following coordinates (in mm) relative to bregma: anterior–posterior = 0.8, medial–lateral = 2.2–2.3 (on either side) and dorsal–ventral = 3.0–3.2. A volume of 500 nl (titer of 1 × 10^9^–2 × 10^9^ GC per ml) was delivered at a rate of 50 nl per pulse with a Nanoject II pressure injection system. Before incision, the animal was injected with Bupivacaine (2–6 mg kg^−1^) and, after injection, the animal was injected with ketofen (2–5 mg kg^−1^) and lactated Ringer’s solution (up to 1 ml) to provide analgesia. Mice were killed at 1–2 months after injection and transcardially perfused with 1× PBS followed by 4% PFA; the brains were dissected for further analysis.

#### Tissue processing for slide-based epifluorescence imaging

Mice were anaesthetized with isoflurane and perfused transcardially with 10 ml of 0.9% saline, followed by 50 ml of 4% PFA. The brain was removed, bisected along the midsagittal plane, placed in 4% PFA overnight and subsequently moved to a 30% sucrose solution until sectioning. Next, 30-μm sections were obtained using a freezing, sliding microtome. Midsagittal sections were collected and stained with DAPI and/or propidium iodide to label nuclei and reveal cellular profiles, respectively. Stained tissue sections were slide mounted using Vectashield hardset mounting medium (Vector Laboratories, H-1400-10) and allowed to dry for 24 h protected from light. Once the mounting medium hardened, the slides were scanned with Aperio VERSA Brightfield epifluorescence microscope (Leica) in the UV, green and red channels, illuminated with a metal halide lamp.

#### Immunohistochemistry

Brain slices were fixed in 4% PFA in PBS at 4 °C overnight or up to 48 h and then transferred to 1× PBS with 0.01% sodium azide as a preservative. Fixed slices were thoroughly washed with PBS to remove residual fixative and then blocked for 1 h at room temperature in 1× PBS containing 5% normal goat serum and 0.2% Triton X-100. After blocking, slices were incubated overnight at 4 °C in blocking buffer containing primary antibodies chicken IgY anti-GFP (Aves Labs, GFP-1020, RRID:AB_10000240; dilution 1:2,000) and mouse anti-ChAT IgG1 for Chol cells (Atlas Labs, AMAb91129, RRID: AB_2665811; dilution 1:500). Following the overnight incubation, slices were washed for 15 min three times with 1× PBS and then incubated for 1 h at room temperature in dye-conjugated secondary antibodies including goat anti-chicken IgY (H + L) Alexa Fluor 488 (Invitrogen, A-11039, RRID:AB_2534096; dilution 1:1,000) and goat anti-mouse IgG1 cross-adsorbed secondary antibody, Alexa Fluor 647 (Invitrogen, A-21240, RRID: AB_2535809; dilution 1:1,000). These antibodies were previously validated for free-floating mouse brain slice immunofluorescence staining, imaging and quantification in the same brain region and cell type^58^. Slices were washed for 15 min three times with 1× PBS, followed by 5 μg ml^−1^ DAPI nuclear staining for 15 min. The slices were then dried on glass microscope slides and mounted with Fluoromount-G (SouthernBiotech). Slides were stored at room temperature in the dark before imaging. Whole-slice montage images were acquired with NIS-Elements imaging software on a Nikon Eclipse Ti2 inverted microscope system equipped with a motorized stage and epifluorescence illumination with standard DAPI, FITC, TRITC, Cy3 and Cy5 excitation and emission filter cubes.

Specificity of enhancer activity for the target striatal cell subclass or type was quantified as the fraction of reporter-antibody-positive neurons that were also marker -antibody-positive. The main region of interest (ROI) for analysis was in the center of the dSTR region and included a minimum of 100 neurons. Completeness of labeling was quantified as the fraction of marker-antibody-positive neurons that were were also reporter-antibody-positive.

#### In vitro electrophysiology and optogenetic stimulation

Enhancer AAV-injected mice were deeply anaesthetized by intraperitoneal administration of Avertin (20 mg kg^−1^) and perfused through the heart with carbogenated (95% O_2_/5% CO_2_) artificial cerebrospinal fluid (aCSF) consisting of 92 mM *N*-methyl-D-glucamine (NMDG), 2.5 mM KCl, 1.25 mM NaH_2_PO_4_, 30 mM NaHCO_3_, 20 mM HEPES, 25 mM glucose, 2 mM thiourea, 5 mM sodium ascorbate, 3 mM sodium pyruvate, 0.5 mM CaCl_2_·4H_2_O and 10 mM MgSO_4_·7H_2_O. Brains were sliced at 300-μm thickness on a vibratome (VT1200S, Leica Biosystems or Compresstome VF-300, Precisionary Instruments) using a zirconium ceramic blade and following the NMDG protective recovery method^96^. Mouse brains were sectioned in the coronal plane such that the angle of slicing was perpendicular to the pial surface. The slices were then transferred to a warmed (32–34 °C) initial recovery chamber filled with NMDG aCSF under constant carbogenation. After 12 min, slices were transferred to a chamber containing HEPES holding aCSF solution consisting of 92 mM NaCl, 2.5 mM KCl, 1.25 mM NaH_2_PO_4_, 30 mM NaHCO_3_, 20 mM HEPES, 25 mM glucose, 2 mM thiourea, 5 mM sodium ascorbate, 3 mM sodium pyruvate, 2 mM CaCl2·4H_2_O and 2 mM MgSO4·7H_2_O, continuously bubbled with 95% O_2_/5% CO_2_. Slices were held in this chamber until used in acute patch-clamp recordings or until fixed in 4% PFA for later histological processing.

Brain slices were placed in a submerged, heated (32–34 °C) chamber that was continuously perfused with fresh, carbogenated aCSF consisting of 119 mM NaCl, 2.5 mM KCl, 1.25 mM NaH_2_PO_4_, 24 mM NaHCO_3_, 12.5 mM glucose, 2 mM CaCl_2_·4H_2_O, 2 mM MgSO_4_·7H_2_O, 1 mM kynurenic acid and 0.1 mM picrotoxin (pH 7.3–7.4). Neurons were visualized with an upright microscope (Scientifica) equipped with infrared differential interference contrast (IR-DIC) optics. Glass patch-clamp pipettes were pulled to an open tip resistance of 2–6 MΩ when filled with the internal recording solution consisting of 110.0 mM potassium gluconate, 10.0 mM HEPES, 0.2 mM EGTA, 4 mM KCl, 0.3 mM Na_2_-GTP, 10 mM phosphocreatine disodium salt hydrate, 1 mM Mg-ATP, 20 mg ml^−1^ glycogen, 0.5 U per ml RNase inhibitor (Takara, 2313A), 0.5% biocytin and 0.02 mM Alexa 594 or 488 (pH-adjusted to 7.3 with KOH). Whole-cell somatic recordings were acquired using a Multiclamp 700B amplifier and custom acquisition software written in Igor Pro (MIES; https://github.com/AllenInstitute/MIES). Electrical signals were digitized at 50 kHz by an ITC-18 (HEKA) and were filtered at 10 kHz. The pipette capacitance was compensated and the bridge was balanced during the current clamp recordings.

To measure peak photocurrent amplitude of striatal neuron types expressing ChR2-H134R–EYFP versus CoChR–EGFP, the area with maximal native reporter fluorescence was first identified. As labeling appeared as dense fluorescent neuropil rather than clearly discernable individual MSN somata, neurons with a healthy appearance under IR-DIC within this densely fluorescent region were selected for voltage clamp recordings with a holding command set to −70 mV (or −60 mV in the case of striatal Chol interneurons). Light was delivered from a mercury arc lamp attached to light guide directed through the ×40 (NA: 0.8) water-immersion microscope objective equipped with a blue excitation spectrum bandpass filter cube. The power density was set to elicit maximal (saturating) photocurrent amplitude (50 mW mm^−2^). To support striatal neuron type identification, in select neurons we recorded in current clamp mode to measure frequency–current curves and signature firing properties.

### Reporting summary

Further information on research design is available in the Nature Portfolio Reporting Summary linked to this article.

## Online content

Any methods, additional references, Nature Portfolio reporting summaries, source data, extended data, supplementary information, acknowledgements, peer review information; details of author contributions and competing interests; and statements of data and code availability are available at 10.1038/s41592-026-03076-z.

## Supplementary information

Supplementary Information Table of author contributions, Supplementary Figs. 1–3 and Tables 1 and 2.

Reporting Summary

Supplementary Video 1 Pattern of light output by a blue emitter.

## Data Availability

The data from this study are available in open repositories under open access licenses. Data from the electrical and optical characterizations (Fig. 2 and Extended Data Figs. 2b–e and 3a,b) are available via Figshare at 10.5522/04/31268383 (ref. ^97^). Data from the experiments demonstrating recording and activation of local neural populations (Fig. 3 and Extended Data Figs. 4 and 5) are available via Zenodo at 10.5281/zenodo.18461445. Data from the spatially resolved neural inactivations (Fig. 4 and Extended Data Figs. 6 and 7) are available via Figshare at 10.6084/m9.figshare.31271761 (ref. ^98^). Data from the subcortical optotagging experiments (Figs. 5 and 6 and Extended Data Figs. 3c–f, 8 and 10) are available via Code Ocean at https://codeocean.allenneuraldynamics.org/capsule/1593868/tree/v1.

## Code availability

The code used in this study is available in open repositories under open access licenses. Code for the electrical and optical characterizations (Fig. 2 and Extended Data Figs. 2b–e and 3a,b) is available via Figshare at 10.5522/04/31268383 (ref. ^97^). Code for the experiments demonstrating recording and activation of local neural populations (Fig. 3 and Extended Data Figs. 4 and 5) is available via Zenodo at 10.5281/zenodo.18461445 (ref. ^99^). Code for the spatially resolved neural inactivations (Fig. 4 and Extended Data Figs. 6 and 7) is available via Figshare at 10.6084/m9.figshare.31271761 (ref. ^98^). Code for the subcortical optotagging experiments (Figs. 5 and 6 and Extended Data Figs. 3c–f, 8 and 10) is available via Code Ocean at https://codeocean.allenneuraldynamics.org/capsule/1593868/tree/v1.
