# Supplementary material for: Neuropixels Opto: combining high-resolution electrophysiology and optogenetics
Source: Nat Methods. 2026 Jun 1;23(6):1207–16. doi: 10.1038/s41592-026-03076-z (PMC13259958; doi:10.1038/s41592-026-03076-z)
Supplement: Supplementary file 1 — Table of author contributions, Supplementary Figs. 1–3 and Tables 1 and 2. [file 41592_2026_3076_MOESM1_ESM.pdf]

nature methods

Supplementary information https://doi.org/10.1038/s41592-026-03076-z

Neuropixels Opto: combining high-resolution electrophysiology and optogenetics

In the format provided by the authors and unedited

Contents

Author contributions 2
Supplementary Figures 3
Supplementary Figure 1 3
Supplementary Figure 2 4
Supplementary Figure 3 5
Supplementary Tables 6
Supplementary Table 1 6
Supplementary Table 2 7

Author contributions

	Bowen	Carandini	Chen	Colonell	Doshi	Dutta	Harris	Häusser	Karsh	Koch	Krumin	Kulik	Ladd	Lakunina	Li	Neutens	O'Callaghan	Olsen	Putzeys	Reddy	Siegle	Socha	Steinmetz	Svoboda	Tilmans	Ting	Vargas	Welkenhuysen	Ye
Conceptualization		●				●	●														●		●	●					
Data curation													●	●		●						●							
Formal analysis											●		●	●							●	●				●	●		
Funding acquisition		●					●	●		●											●		●	●					
Investigation	●		●								●		●	●	●	●		●				●				●	●		●
Methodology						●					●					●	●		●	●	●	●		●	●	●		●	
Project administration		●				●															●								
Resources		●				●	●														●		●	●		●			
Software				●	●				●		●	●									●								
Supervision		●				●	●	●		●											●		●	●		●			
Validation				●					●													●							
Visualization		●											●	●							●	●				●			
Writing – original draft		●											●								●	●	●						
Writing –editing		●											●	●		●					●	●	●	●					

Author contributions according to the CRediT taxonomy.

Supplementary Figures

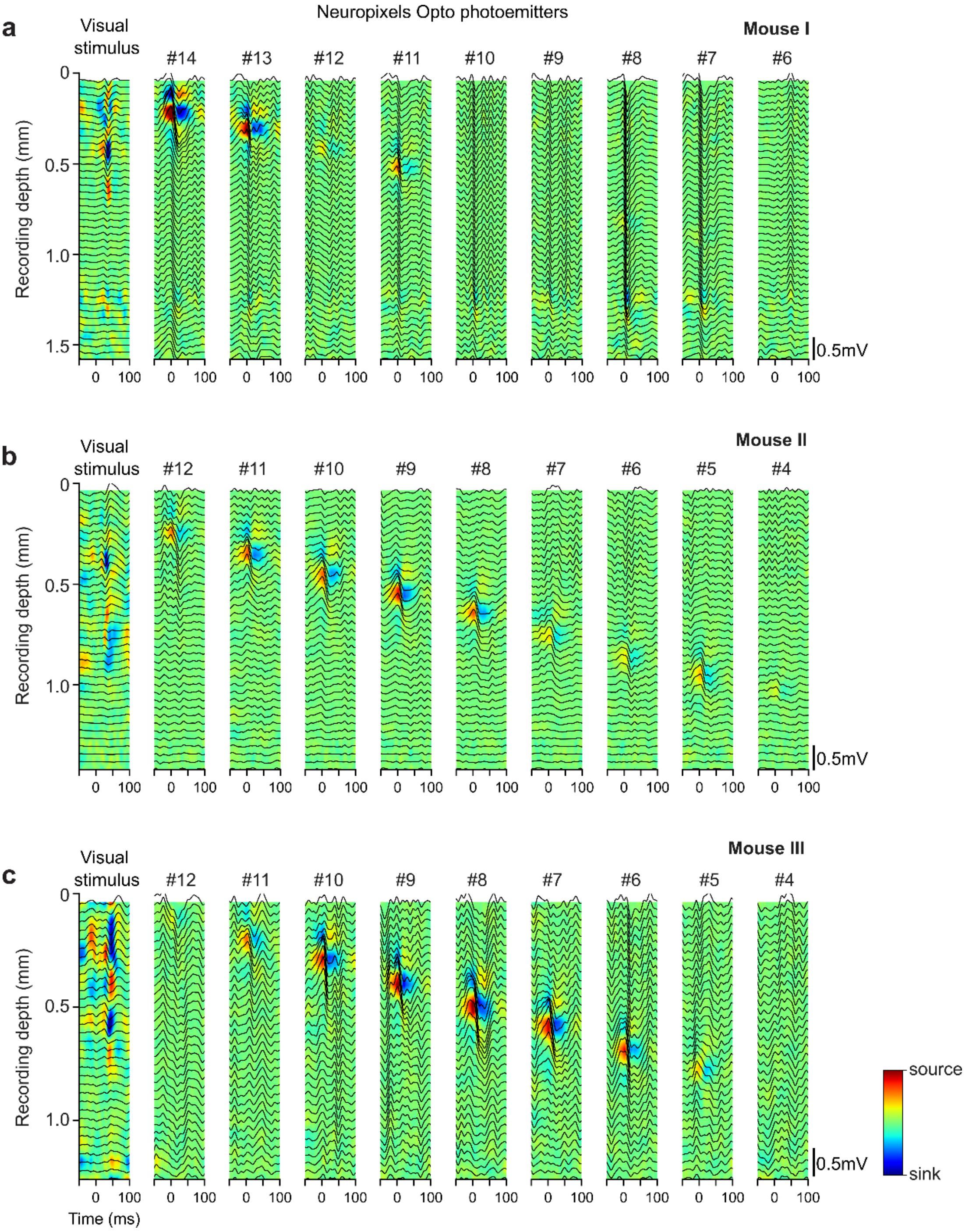

Supplementary Figure 1

Field potentials elicited by the emitters. Local field potentials (LFPs) and current source density (CSD) profiles of activity elicited by the emitters in the primary visual cortex of mice expressing ChRmine, for the three experiments highlighted in **Figure 3**. LFPs were computed by bandpass filtering the signal (5–60 Hz) using non-causal, zero-phase delay filtering; averaging across 40 trials; and aligning to the stimulus onsets. For visual stimulation, these onsets were contrast reversals of the checkerboard stimulus (*leftmost column*). For emitters, the onsets were relative to one of 9 emitters starting at the top of the cortex (*remaining columns*). CSD profiles show current sinks (blue, inward currents) and sources (red, outward currents), indicating neural activation specific to the emitter location. The field potentials in response to the surface laser contained artifacts and were not analyzed. **a**. Mouse I (session 1). **b.** Mouse II (session 3). **c**. Mouse III (session 3).

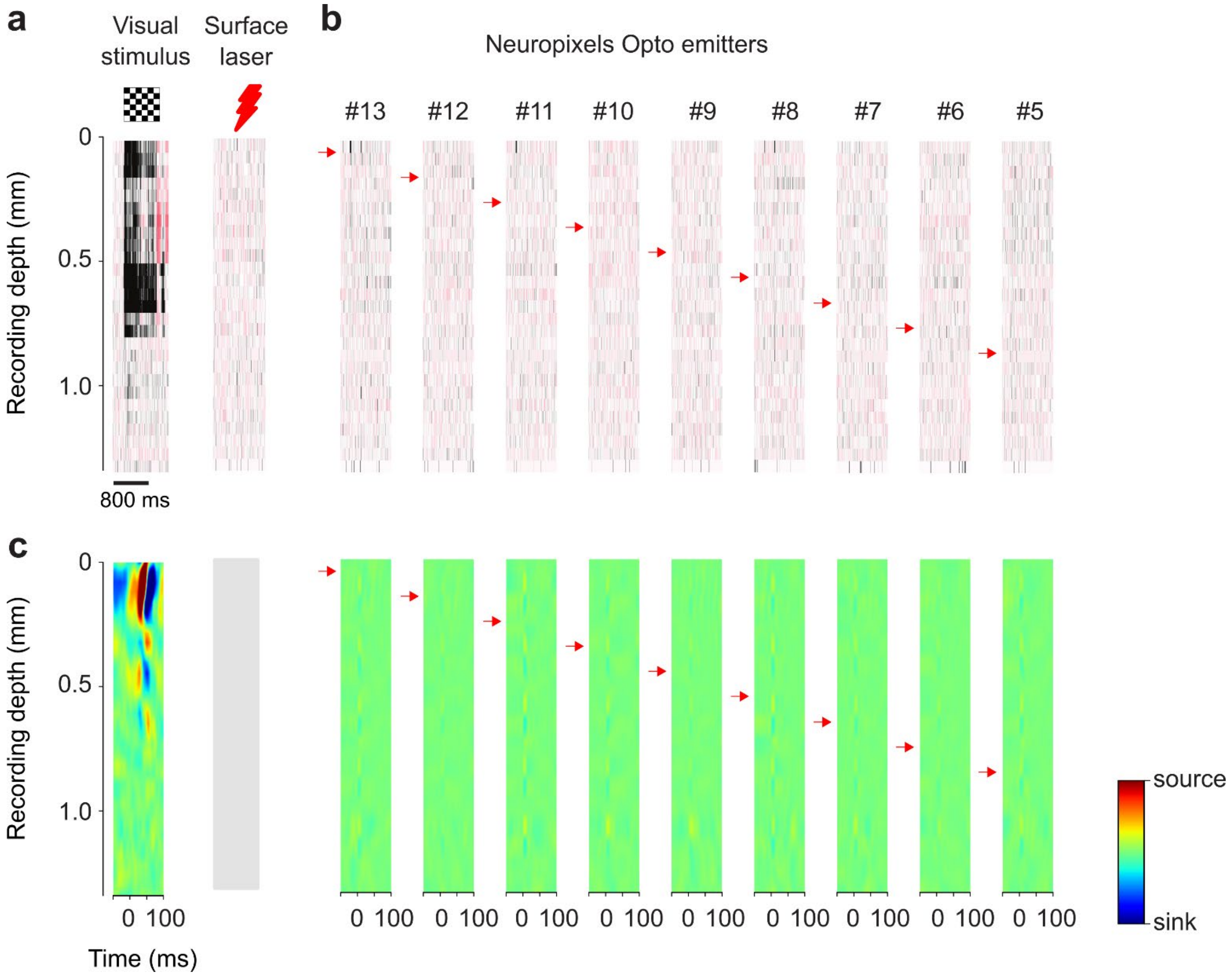

Supplementary Figure 2

Control recordings in a mouse without opsin. The same experiment as in Figure 3 was conducted in a control mouse that expressed no opsin. As expected, light stimuli (external or via the emitters) did not elicit any activity. **a-b**. Firing rate as a function of depth in response to visual stimulation, external laser, and stimulating emitters Format as in **Figure 3d-e**. **c**. Local field potentials and current source densities measured in the same experiments. Format as in **Figure S9**. The field potentials in response to the surface laser (*gray rectangle*) contained artifacts and were not analyzed.

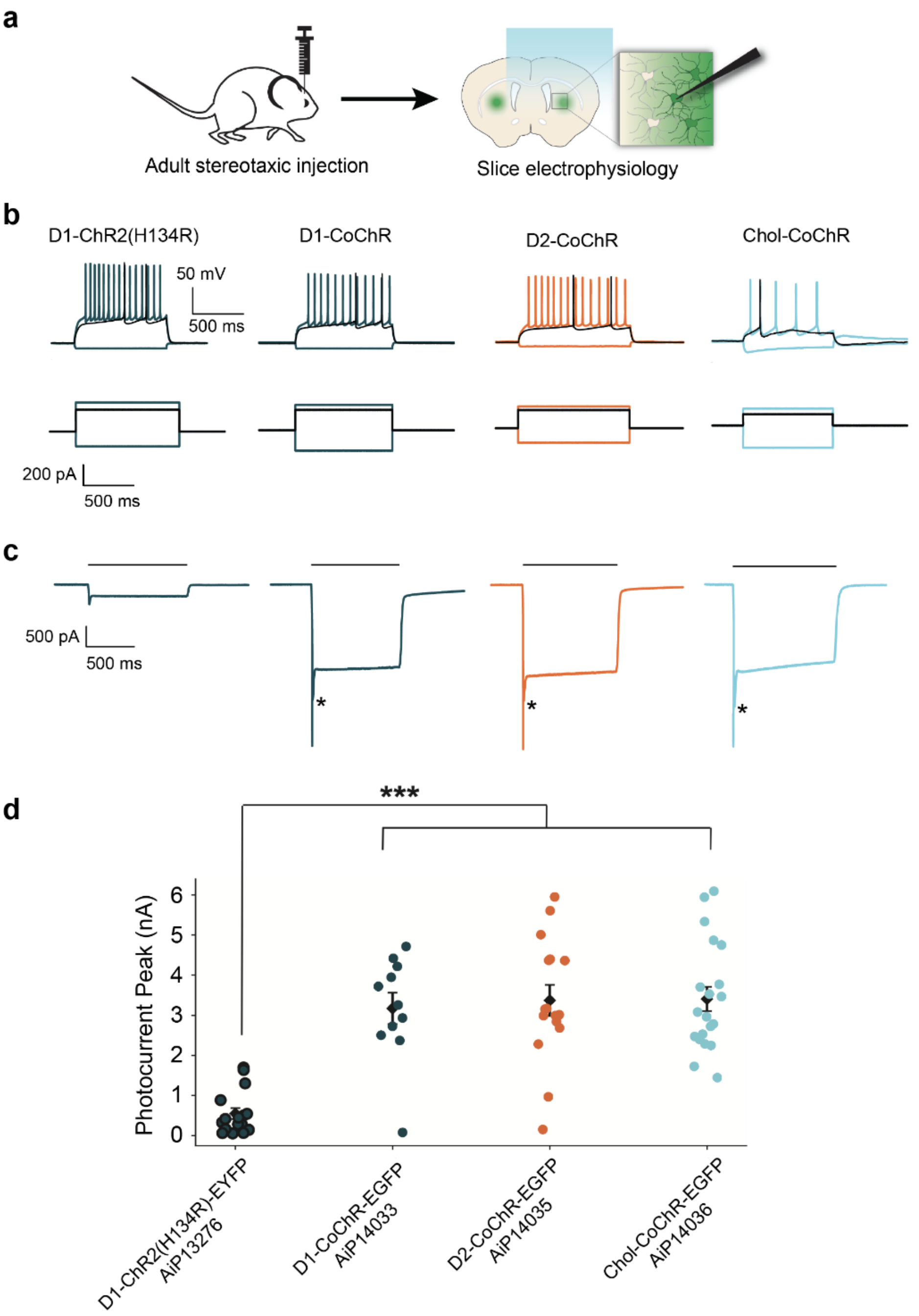

Supplementary Figure 3

Photocurrents measured in acute brain slice patch-clamp recordings. **a**. Following the injection of AAV vectors into mouse brain (*left*) we performed acute brain slice recordings with blue light stimulation in the dorsal striatum (*right*). **b**. Whole-cell current clamp recordings in 4 representative opsin-expressing neurons, in response to a series of 1 s current injection steps (*bottom*). Putative cell type and expressed opsin are indicated. **c**. Whole-cell voltage clamp recordings from the same representative neurons, showing photocurrents evoked by 1 s blue light stimulation (*bars*). Light intensity was adjusted in each case to achieve maximal photocurrents. Asterisks denote photocurrent peak amplitude which is obscured by escape action currents observed in the CoChR-EGFP groups. **d**. Summary of peak photocurrent amplitude measurements (D1-ChR2(H134R)-EGFP, N = 20 cells; D1-CoChR-EGFP, N = 11 cells; D2-CoChR-EGFP, N = 18 cells; Chol-CoChR-EGFP, N = 21 cells). Welch's ANOVA revealed a significant difference for mean photocurrent amplitudes across groups, and post-hoc multiple comparison test revealed that all CoChR-EGFP groups were significantly different from the D1-ChR2(H134R)-EYFP group ($p < 0.0005$, ***).

Supplementary Tables

Region	Cell type	Opsin	Fluorophore	# Optotagged	Sessions	Mice
CP	D1 MSN	ChRmine	mScarlet	7	2	1
CP	D1 MSN	CoChR	EGFP	117	17	10
CP	D1 MSN	rsChRmine	oScarlet	1	1	1
CP	D2 MSN	ChRmine	mScarlet	8	3	1
CP	D2 MSN	somBiPOLES	mCerulean	2	2	1
CP	D2 MSN	ChrimsonR	tdTomato	4	1	1
CP	D2 MSN	CoChR	EGFP	60	10	7
CP	Chol	ChRmine	mScarlet	17	6	4
CP	Chol	somBiPOLES	mCerulean	1	1	1
CP	Chol	CoChR	EGFP	1	1	1
CP	Chol	rsChRmine	oScarlet	7	2	1
GPe	Ntrk1	rsChRmine	oScarlet	10	3	3
MRN	Vglut2	ChRmine	mScarlet	6	1	1
MRN	Gad67	ChRmine	mScarlet	20	4	3
**Total**				**261**	**40***	**26***

Supplementary Table 1

Regions and cell types for subcortical optotagging. Details of optotagged units included in **Figure 5**. The number of units optotagged per session for a given cell type ranges between 1 and 21. Sessions may appear multiple times in this table as multiple cell types were tagged per session. * Because a session / mouse can appear in two rows, the total number of sessions and mice is in the last row is lower than the sum of the individual rows.

	Target (2019)	Result (2023)
Shank length	10 mm	10 mm
Shank width	70 µm	70 µm
Shank thickness	≤ 35 µm	33 µm
Number of shanks	1	1
Silicon Base area (W_b x L_b)	7x9 mm	9.6 x 10.2 mm
Package base thickness	≤ 1 mm	1.1 mm
Maximum bow: base to tip	≤ ± 200 µm	≤ ± 200 µm
Emission wavelength (Blue)	450-470 nm	450 nm
Emission wavelength (Amber/Red)	570-650 nm	638 nm
Emitter dimension (W x L)	15 x 35 µm	0.45 x 32 µm (blue)<br>0.60 x 42 µm (red)
Emitter emission patterns	Hemispherical, collimated, or focused	See Figure 2
Emitter pitch	200 µm	100 µm
Emitter intensity (100 µm from shank)	2 mW/mm^2 @470nm	See Figure 2
Emitter switching speed	< 1 ms	~12 µs *
Emitter count (sites/color)	14	14
Electrode material	TiN	TiN
Electrode dimension	12 x 12 µm	12 x 12 µm
Electrode pitch	15 to 20 µm	20 µm
Electrode count	300-500	960
Channel count	150-300	384
Thermal dissipation during use (difference between probe surface and tissue)	< 1ºC	< 1ºC

Supplementary Table 2

Device specifications for Neuropixels Opto probes. For each specification, we provide the value or range sought in 2019 before the design of the probe ("Target") and the value obtained after designing and testing the probes in 2023 ("Result"). *The emitter switching speed indicates the physical limits (for a transition from 10% to 90%), but the software control in the current version of the probe is not real-time, so the switching speed visible to a user is much longer (multiple milliseconds).
