# Supplementary material for: Neuropixels Opto: combining high-resolution electrophysiology and optogenetics
Source: Nat Methods. 2026 Jun 1;23(6):1207–16. doi: 10.1038/s41592-026-03076-z (PMC13259958; doi:10.1038/s41592-026-03076-z)
Supplement: Supplementary file 2 — Reporting Summary [file 41592_2026_3076_MOESM2_ESM.pdf]

nature portfolio

Corresponding author(s): Josh Siegle and Matteo Carandini

Last updated by author(s): Feb 5, 2026

Reporting Summary

Nature Portfolio wishes to improve the reproducibility of the work that we publish. This form provides structure for consistency and transparency in reporting. For further information on Nature Portfolio policies, see our Editorial Policies and the Editorial Policy Checklist.

Statistics

For all statistical analyses, confirm that the following items are present in the figure legend, table legend, main text, or Methods section.

n/a	Confirmed	
☐	☒	The exact sample size (*n*) for each experimental group/condition, given as a discrete number and unit of measurement
☐	☒	A statement on whether measurements were taken from distinct samples or whether the same sample was measured repeatedly
☐	☒	The statistical test(s) used AND whether they are one- or two-sided<br>*Only common tests should be described solely by name; describe more complex techniques in the Methods section.*
☐	☒	A description of all covariates tested
☒	☐	A description of any assumptions or corrections, such as tests of normality and adjustment for multiple comparisons
☐	☒	A full description of the statistical parameters including central tendency (e.g. means) or other basic estimates (e.g. regression coefficient) AND variation (e.g. standard deviation) or associated estimates of uncertainty (e.g. confidence intervals)
☐	☒	For null hypothesis testing, the test statistic (e.g. *F*, *t*, *r*) with confidence intervals, effect sizes, degrees of freedom and *P* value noted<br>*Give P values as exact values whenever suitable.*
☒	☐	For Bayesian analysis, information on the choice of priors and Markov chain Monte Carlo settings
☒	☐	For hierarchical and complex designs, identification of the appropriate level for tests and full reporting of outcomes
☒	☐	Estimates of effect sizes (e.g. Cohen's *d*, Pearson's *r*), indicating how they were calculated

Our web collection on statistics for biologists contains articles on many of the points above.

Software and code

Policy information about availability of computer code

Data collection	SpikeGLX (https://billkarsh.github.io/SpikeGLX/)<br>Open Ephys GUI (https://open-ephys.org/gui)<br>WavPack (https://www.wavpack.com/)
Data analysis	Matlab<br>Python<br>Kilosort 2.5 (https://zenodo.org/records/4482749)<br>Kilosort 4.0 (https://zenodo.org/records/15265341)<br>SpikeInterface (https://spikeinterface.readthedocs.io/en/stable/)<br>spks (https://github.com/spkware/spks)<br>Neuropyxels (https://github.com/m-beau/NeuroPyxels)<br>Nextflow (https://www.nextflow.io/)<br>Code Ocean (https://codeocean.com/)<br>UnitRefine (https://figshare.com/articles/software/UnitRefine/28282841)

For manuscripts utilizing custom algorithms or software that are central to the research but not yet described in published literature, software must be made available to editors and reviewers. We strongly encourage code deposition in a community repository (e.g. GitHub). See the Nature Portfolio guidelines for submitting code & software for further information.

Data

Policy information about availability of data

All manuscripts must include a data availability statement. This statement should provide the following information, where applicable:

- Accession codes, unique identifiers, or web links for publicly available datasets
- A description of any restrictions on data availability
- For clinical datasets or third party data, please ensure that the statement adheres to our policy

The data from this study are available in open repositories under open access licenses.
Data from the electrical and optical characterizations (Figure 2, Extended Data Fig 2b-e, Extended Data Fig 3a,b) are available at https://figshare.com/s/85fb292e753f5d45a7e6.
Data from the experiments demonstrating recording and activation of local neural populations (Figure 3, Extended Data Figs 4 and 5) are available at https://doi.org/10.5281/zenodo.18461445
Data from the spatially resolved neural inactivations (Figure 4 and Extended Data Figs 6 and 7) are available at UW link here.
Data from the subcortical optotagging experiments (Figures 5 and 6 and Extended Data Figs 3c-f, 8 and 10) are available at https://codeocean.allenneuraldynamics.org/capsule/3147084/tree/v1.

Research involving human participants, their data, or biological material

Policy information about studies with human participants or human data. See also policy information about sex, gender (identity/presentation), and sexual orientation and race, ethnicity and racism.

Reporting on sex and gender	N/A
Reporting on race, ethnicity, or other socially relevant groupings	N/A
Population characteristics	N/A
Recruitment	*Describe how participants were recruited. Outline any potential self-selection bias or other biases that may be present and how these are likely to impact results.*
Ethics oversight	N/A

Note that full information on the approval of the study protocol must also be provided in the manuscript.

Field-specific reporting

Please select the one below that is the best fit for your research. If you are not sure, read the appropriate sections before making your selection.

☒ Life sciences ☐ Behavioural & social sciences ☐ Ecological, evolutionary & environmental sciences

For a reference copy of the document with all sections, see nature.com/documents/nr-reporting-summary-flat.pdf

Life sciences study design

All studies must disclose on these points even when the disclosure is negative.

Sample size	N/A. Our results do not rest on statistical tests, so we do not need to select a sample size in advance We simply recorded from vast numbers of neurons in multiple sessions and in multiple mice, and observed similar results.
Data exclusions	N/A. No data were excluded.
Replication	As shown in the paper, results were very similar across sessions and across mice.
Randomization	Stimulus conditions were randomized
Blinding	Not relevant to our study: there aren't multiple treatments, etc.

Reporting for specific materials, systems and methods

We require information from authors about some types of materials, experimental systems and methods used in many studies. Here, indicate whether each material, system or method listed is relevant to your study. If you are not sure if a list item applies to your research, read the appropriate section before selecting a response.

Materials & experimental systems

n/a	Involved in the study
☐	☒ Antibodies
☒	☐ Eukaryotic cell lines
☒	☐ Palaeontology and archaeology
☐	☒ Animals and other organisms
☒	☐ Clinical data
☒	☐ Dual use research of concern
☒	☐ Plants

Methods

n/a	Involved in the study
☒	☐ ChIP-seq
☒	☐ Flow cytometry
☒	☐ MRI-based neuroimaging

Antibodies

Antibodies used

Primary antibodies:
chicken anti-GFP IgY antibody (Vendor: Aves Labs, Cat# GFP-1020, RRID:AB_10000240, dilution 1:2000)
mouse anti-ChAT IgG1 antibody (Vendor: Atlas Labs, Cat# AMAb91129, RRID: AB_2665811, dilution 1:500)
Secondary antibodies:
Goat anti-Chicken IgY (H+L) Alexa Fluor™ 488 (Vendor: Invitrogen, Cat# A-11039, RRID:AB_2534096, dilution 1:1000)
Goat anti-Mouse IgG1 Cross-Adsorbed Secondary Antibody, Alexa Fluor™ 647 (Vendor: Invitrogen, Cat# A-21240, RRID: AB_2535809, dilution 1:1000)

Validation

In vivo validation for free-floating mouse brain slice immunfluorescence staining, imaging, and quantification in the same brain region and cell type was performed at the Allen Institute. Exemplary data has been previously published in Hunker et al, Neuron 2025.

Animals and other research organisms

Policy information about studies involving animals; ARRIVE guidelines recommended for reporting animal research, and Sex and Gender in Research

Laboratory animals

species strain and age
Activating local neural populations (UCL): 2 wildtype mice (C57BL/6), 10-16 weeks
Activating local neural populations (UCL): 2 double transgenic mice (Ai3269 x PV-Cre70), 10-16 weeks
Driving local circuits (UW): 3 transgenic mice (CaMK2a-tTA.tetO-G8s), 19 weeks
Optotagging nearby neurons (AI): 26 transgenic mice, 11-28 weeks as follows:
• Chat-IRES-Cre83 (JAX #031661)
• Chat-IRES-Cre-neo83 (JAX #006410)
• Sst-IRES-Cre (JAX #028864)
• Drd1a-Cre84 (JAX #037156)
• Adora2a-Cre (MMRRC #36158)
• Slc17a6-IRES-Cre85 (JAX #028863)
• Ntrk1-IRES-Cre (MMRRC #15500)
• Gad2-IRES-Cre86 (JAX #028867)

Wild animals

N/A (no wild animals)

Reporting on sex

Activating local neural populations: 2 males 2 females

Field-collected samples

N/A (no field-collected samples)

Ethics oversight

UCL: oversight provided by the Home Office according to the UK Animals Scientific Procedures Act (1986)
UW: protocols approved by the Institutional Animal Care and Use Committee (IACUC)
AI: protocols approved by the Institutional Animal Care and Use Committee (IACUC)

Note that full information on the approval of the study protocol must also be provided in the manuscript.

Plants

Seed stocks	N/A
Novel plant genotypes	N/A
Authentication	N/A
